# Evidence for Involvement of Wnt Signalling in Body Polarities, Cell Proliferation, and the Neuro-Sensory System in an Adult Ctenophore

**DOI:** 10.1371/journal.pone.0084363

**Published:** 2013-12-31

**Authors:** Muriel Jager, Cyrielle Dayraud, Antoine Mialot, Eric Quéinnec, Hervé le Guyader, Michaël Manuel

**Affiliations:** Systématique, Adaptation, Evolution, Unité Mixte de Recherche (UMR) 7138 CNRS (Centre National de la Recherche Scientifique), Université Pierre et Marie Curie – Paris 6, Paris, France; Laboratoire de Biologie du Développement de Villefranche-sur-Mer, France

## Abstract

Signalling through the Wnt family of secreted proteins originated in a common metazoan ancestor and greatly influenced the evolution of animal body plans. In bilaterians, Wnt signalling plays multiple fundamental roles during embryonic development and in adult tissues, notably in axial patterning, neural development and stem cell regulation. Studies in various cnidarian species have particularly highlighted the evolutionarily conserved role of the Wnt/β-catenin pathway in specification and patterning of the primary embryonic axis. However in another key non-bilaterian phylum, Ctenophora, Wnts are not involved in early establishment of the body axis during embryogenesis. We analysed the expression in the adult of the ctenophore *Pleurobrachia pileus* of 11 orthologues of Wnt signalling genes including all ctenophore Wnt ligands and Fz receptors and several members of the intracellular β-catenin pathway machinery. All genes are strongly expressed around the mouth margin at the oral pole, evoking the Wnt oral centre of cnidarians. This observation is consistent with primary axis polarisation by the Wnts being a universal metazoan feature, secondarily lost in ctenophores during early development but retained in the adult. In addition, local expression of Wnt signalling genes was seen in various anatomical structures of the body including in the locomotory comb rows, where their complex deployment suggests control by the Wnts of local comb polarity. Other important contexts of Wnt involvement which probably evolved before the ctenophore/cnidarian/bilaterian split include proliferating stem cells and progenitors irrespective of cell types, and developing as well as differentiated neuro-sensory structures.

## Introduction

The Wnts are a metazoan-specific family of extracellularly secreted signal proteins which play central roles in the regulation of cell behaviour and fate, notably during embryonic development and in the control of tissue homeostasis during adult life [1]–[3]. Wnt genes known in bilaterian animals have been classified in 13 distinct subfamilies [4], [5]. Most of the duplications that generated this diversity of ligands occurred before the cnidarian/bilaterian split [4], whereas Wnts appear to be much less diversified in the genomes of the sponge *Amphimedon queenslandica* [6], [7] and of the ctenophore *Mnemiopsis leidyi* [8].

Wnt ligands classically signal through binding to Frizzled (Fz) membrane receptors, and activated Fz proteins can elicit one or several of distinct Wnt pathways (depending on cellular state) each corresponding to a specific cascade of intracellular events [9], [10]. In the Wnt/β-catenin pathway (also known as the canonical Wnt pathway), upon activation Fz and its co-receptor LRP5/6 recruit the cytoplasmic protein Dishevelled (abbreviated as Dvl or Dsh), which binds a complex formed by Axin, GSK-3β (glycogen synthase kinase-3β) and APC (adenomatous polyposis coli). As a result, GSK-3β is phosphorylated and inactivated. This blocks the constitutive degradation of cytoplasmic β-catenin (β-cat) and thus allows its accumulation in the nucleus where it associates with the transcription factor TCF/LEF to activate specific target genes. Alternative (“non-canonical”) Wnt signalling pathways use downstream cascades independent from β-catenin, the Axin/GSK-3β/APC complex and TCF, but yet often involve Fz receptors and their cytoplasmic partner Dvl like in the canonical pathway. One of these “non-canonical” Wnt pathways, termed Wnt/PCP (for planar cell polarity), is thought to work either through Rho family GTPases or JNK regulatory modules, but might in fact represent two distinct pathways (Fz-PCP and Fz-Rho, [11]). In another β-catenin independent pathway (Wnt/Ca^2+^), Wnts can bind to non-Fz receptors (Ryk) and intracellular effects are triggered by an increase of Ca^2+^ release to the cytoplasm caused by activation of phospholipase-C (PLC) [10].

Wnt/β-catenin is the most intensively studied of the Wnt pathways, particularly in a developmental context in bilaterian models (e.g. vertebrates and *Drosophila*), where it plays multiple key roles. These include primary axis establishment during development [12], [13], regulation of stem cell proliferation, self-renewal and pluripotency [9], [14], morphogenesis in multiple contexts such as tooth development in mammals [15] or feather formation in birds [16], and brain development and function [10], [13]. There is also quite a large amount of data on the expression and function of the Wnt/β-catenin pathway in cnidarians, mainly concerning its roles in axial polarity, gastrulation and endoderm specification, and stem cell regulation ([17]–[28] and many other references).

**Figure 1:**
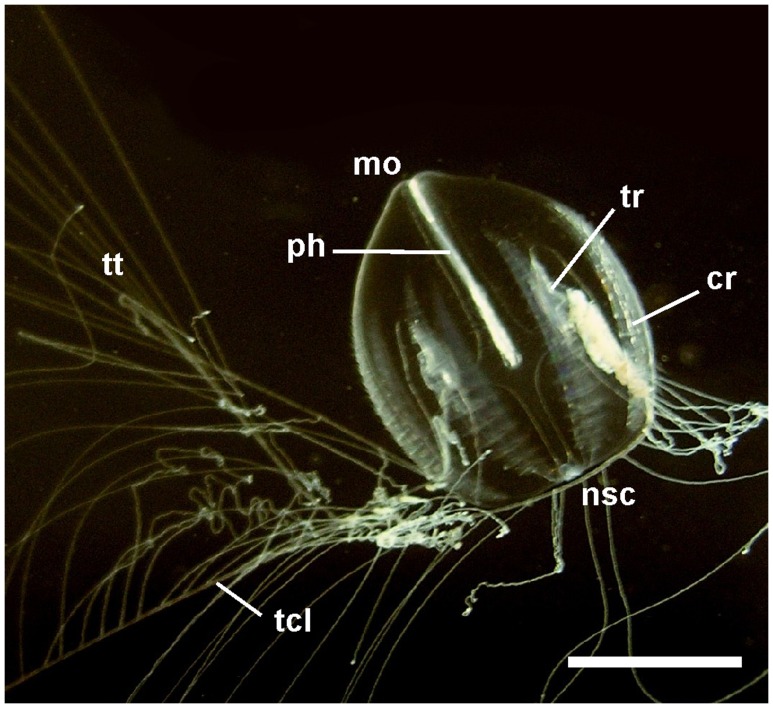
General morphology of the ctenophore *Pleurobrachia pileus*. The mouth (mo) and the aboral neuro-sensory complex (nsc) are located respectively at the oral and aboral pole of the single body axis. The eight comb rows (cr) are locomotory structures, each made of regularly repeated swimming paddles or combs. The body is biradially symmetrical notably due to the presence of a single pair of tentacles (tcl), each bearing numerous lateral branchings or tentillae (tt). The tentacle root (tr), enclosed in the tentacle sheath, is responsible for constant regeneration of tentacle tissues. The pharynx (ph) and some canals of the endodermal gastro-vascular system are also visible by transparency. Scale bar: 1 cm.

Several recent studies have emphasised the interest of ctenophores (Fig. 1), one of the four “early-diverging” (i.e. non-bilaterian) animal phyla, to address the ancient evolutionary history of fundamental animal characteristics, such as body plan properties and underlying regulatory mechanisms, stem cell genes, or the origin of muscles and the nervous system (e.g. [8], [29]–[36]). A genomic survey of Wnt/β-catenin pathway genes in the ctenophore *M. leidyi* [8] identified most of the known components of this pathway (including *bona fide* Wnt ligands, Fz receptors, LRP5/6, Dvl, GSK-3β, β-cat and TCF). The Wnt family is nevertheless poorly diversified in ctenophore with only four members. Furthermore, expression of these Wnt genes during embryonic development could not be detected until well after the oral/aboral axis becomes phenotypically apparent, suggesting that unlike in bilaterians, cnidarians and sponges [12], [13], [19], [37], [38], Wnt signalling in ctenophore probably plays no role in setting up the primary body axis.

Interpretation of these observations in evolutionary terms is problematic because the branching order of the non-bilaterian phyla remains strongly debated. Some phylogenomic analyses have placed ctenophores as the sister group to all other extant metazoans [39], [40], a result probably due to a long-branch attraction artifact [41]–[43]. In other phylogenomic studies [41], [42], [44], ctenophores formed with cnidarians a “coelenterate” clade, grouped with bilaterians in the Eumetazoa (animals with nerve and muscle cells), while the other two non-bilaterian phyla, sponges and placozoans, branched outside this eumetazoan clade with otherwise unresolved positions. The proposed sister-group relationship between ctenophores and cnidarians implies however a rather high number of gene losses in the ctenophore lineage, including of many Wnt ligand subfamilies [8] and of several other developmental genes (e.g. some homeobox gene families [31]) as well as gene function modifications, which are not simple to reconcile.

The adult ctenophore *Pleurobrachia pileus* is a particularly suited experimental model to investigate the contribution of Wnt signalling to the body plan features typical of this phylum, in particular in the adult, since there is detailed information available concerning anatomy and cellular dynamics of adult tissue renewal for this species. This includes the recent characterisation of multiple localised populations of somatic stem cells [32], with instances of orderly progression of cellular lineages along “cellular conveyor belts” [32], [34], [45], as well as a recent re-description using immunohistochemistry of the architecture of the neuro-sensory system, shown to be much more complex than traditionally thought [46].

*Pleurobrachia pileus* is a marine animal like all ctenophores and lives in the plankton like most other members of the phylum. It displays all characteristic features of the highly original and complex ctenophore body plan, including biradial symmetry (for definition see [38]) and a locomotory system consisting of eight distinctive meridional rows of swimming paddles called combs (Fig. 1), each made of the many fused giant cilia of “polster cells”. At their aboral pole, ctenophores possess an apical sensory organ involved in equilibration and flanked by two elongated ciliated areas called polar fields. There are two distinct nerve nets extending throughout the body, the epithelial (or polygonal) nerve net and the mesogleal nerve net, the former giving rise to specialised condensations in several regions of the body surface [46]. The complex gastro-vascular system, of mainly endodermal origin, opens at one extremity by the mouth and at the other by two anal pores. *Pleurobrachia pileus* like most ctenophores catches preys using a pair of long and contractile tentacles which bear lateral branches or tentillae on their oral side (Fig. 1). The epidermis of tentacles and tentillae is densely covered with adhesive cells called colloblasts, which stick to the prey. Tentacles can extend from and retract into a tentacular sheath in which the tentacle root is housed.

A *P. pileus* transcriptomic assembly was used to identify the main Wnt signalling genes previously characterised by Pang *et al.* [8] in *M. leidyi*. Gene *in situ* hybridisation (ISH) using antisense RNA probes were performed for 11 genes: the four ctenophore Wnt ligands (named *PpiWnt6, PpiWnt9, PpiWntA, PpiWntX* according to [8]), the two ctenophore Fz receptors (*PpiFzA* and *PpiFzB*), the two identified paralogues of *Dvl*, and the *P. pileus* orthologues of GSK-3β, β-cat and TCF. Among these genes, Wnt ligands, Fz receptors, and Dvl proteins are upstream components shared by several signalling pathways. Fz and Dvl proteins are also thought to function in the so-called Fz-PCP pathway independently from both Wnt ligands and the downstream canonical machinery [11]. Likewise the β-catenin protein has multiple cellular functions besides its role in Wnt canonical signalling, notably in cell adhesion complexes [47], and GSK-3β is a highly pleiotropic cytoplasmic enzyme [48], [49]. Thus, identifying from gene expression patterns which Wnt signalling cascade or other transduction pathway is activated downstream of these components is not an easy task. Nonetheless direct or indirect lines of evidence exist to assess at least the activation of the Wnt/β-catenin pathway. Activation of canonical Wnt signalling is usually monitored by checking levels of nuclear vs. cytoplasmic β-catenin, but this requires using a specific anti-β-catenin antibody, not available in *P. pileus*. In this study, we invoke two lines of indirect evidence to identify territories in which Wnts likely activate the β-catenin pathway: (i) synexpression of genes involved in reception of Wnt signal whatever the downstream pathway (*Fz, Dvl*) together with β-catenin pathway genes (*GSK-3, β-cat, TCF*); (ii) more specifically, strong expression of the transcription factor *TCF*. Indeed *TCF* is a good marker of Wnt/β-catenin pathway activation (e.g., experimental evidence in cnidarians for positive feedback of *TCF* transcription upon activation of canonical Wnt signalling [17], [18]).

Along with description of the expression pattern of these 11 Wnt signalling genes, we provide original data on some aspects of *P. pileus* neuro-anatomy and on the dynamics of some adult cellular lineages when deemed useful for interpretation of the gene expression patterns. The observed deployment of Wnt signalling genes within the adult ctenophore body provides support for ancient roles of this signalling pathway in the following contexts: polarity along the main body axis as well as at more local spatial scales; undifferentiated and proliferating cells within all kinds of cellular lineages; and the neuro-sensory system.

## Materials and Methods

### Specimen Collection

Adult specimens of *Pleurobrachia pileus* were collected using plankton nets in Roscoff and in Gravelines (France) during their reproductive season, between March and June. They were transferred into filtered natural seawater and kept at 16°C. The field work done in the context of this work did not involve any endangered or protected species. Animal collects in the field did not require any specific permission.

### Gene Searches and Cloning; Antisense RNA Probe Synthesis

Sequences of most Wnt/β-catenin pathway genes investigated in this study were retrieved and assembled from a *P. pileus* EST collection publicly available in dbEST (http://www.ncbi.nlm.nih.gov/nucest?term=pleurobrachia). Since we store physical replicates of these ESTs in the lab, antisense RNA probes for these genes were produced directly from the corresponding plasmids. Two of the genes (*PpiWnt9* and *PpiWntA*) were instead recovered from an unpublished Newbler assembly from one run of 454 sequencing of total RNA extracted from mixed embryonic, larval, and adult stages (see [34]) and therefore PCR (polymerase chain reaction) amplification and cloning were required for these genes. Two sets of non-degenerate primers per gene were designed and two rounds of PCR amplification were performed using cDNA prepared from adults as template. PCR products were purified using the QIAquick Gel extraction Kit (Qiagen) and cloned into a pGEM®-T Easy Vector (Promega). For all genes, plasmids were sequenced as a routine control prior to RNA probe synthesis. Phylogenetic analyses of the sequences were systematically performed (see procedures and trees in File S1). The gene sequences are available from the GenBank database under the following reference numbers: *PpiWnt6:* FQ001620.1; *PpiWntX*: CU420621.1; *PpiWntA*: KF758439; *PpiWnt9*: KF758438; *PpiFzA*: FQ005841.1; *PpiFzB:* FP991039.1; *PpiDvl1*: CU416561.1; *PpiDvl2*: FQ013569.1; *PpiGSK-3β*: CU418898.1; *Ppiβ-cat*: CU421890.1; *PpiTcf*: FP990243.1.

### Gene *in situ* Hybridisation

Adults were fixed at 4°C in 4% paraformaldehyde in 50% seawater and 50% PBST (10 mM Na_2_HPO4, 150 mM NaCl, pH 7.5, 0.1% Tween 20) for 1 hour then washed three times in PBST and dehydrated through a graded series of ethanol and stored in methanol at −20°C. The *in situ* hybridisation (ISH) protocol was as described in [34]. Following whole-mount ISH, specimens were micro-dissected. Tentacle roots were extracted from the body for separate observation. The aboral region was isolated from the rest of the body and positioned on a slide. Negative controls (with a sense probe and without any RNA probe) performed in parallel showed no staining after extensive revelation, except occasionally in isolated cells of the non-specialised ectodermal epithelium (between adjacent comb rows, around the apical organ). For this reason, staining of isolated epithelial cells occasionally obtained with some probes was not taken into account. Transverse cryo-sectionning of tentacle roots after whole mount ISH was performed using a Leica CM1860 cryostat at a thickness of 14 µm, as described in [32].

### Immunofluorescence Staining of Conserved Proteins and Neurotransmitters

Animals were fixed in 4% paraformaldehyde in PBS for 30 min, at room temperature, then samples were washed several times in PBS, dehydrated through a graded series of ethanol and stored in methanol at −20°C. After stepwise re-hydration to PBS, samples were permeabilised with Triton-X100 (0.2% in PBS, then 0.01% in PBS, 10 min at room temperature). After blocking with 1% bovine serum albumin, samples were incubated with one of the following primary antibodies for 4 hours at room temperature: rat monoclonal anti-tyrosylated α-tubulin (YL1/2, Oxford biotechnology, 1/1000), rabbit polyclonal anti-L Dopa (AB6426, Abcam, 1/500), rabbit polyclonal anti-serotonin (5HT) (AB10385, Abcam, 1/400), rabbit polyclonal anti-norepinephrine (NE) (AB120, Millipore, 1/200), rabbit polyclonal anti-Tyrosine Hydroxylase (TH) (Cell signalling technology, #2792, 1/100) and rabbit polyclonal anti-Vmat2 (kindly provided by Salah el Mestikawy). A rabbit polyclonal anti-phospho-histone H3 antibody (Millipore, 06–570, 1/200) was used to detect mitotic cells as described in [50]. After washing in PBS triton-X100 0.01%, samples were incubated overnight at 4°C with the Alexa Fluor® 568 goat anti-rat IgG and/or Alexa Fluor® 488 goat anti-rabbit IgG (Molecular probes) as secondary antibodies. Nuclei were stained with DAPI (1 µg/ml) for 15 mn in PBST and mounted in Citifluor® solution.

### EdU Labelling of DNA-replicating Cells

EdU incorporation assays were done using the Click-iT® EdU Alexa Fluor® 488 Imaging Kit from Invitrogen (Durham, NC, USA). The protocol was as described in [32].

### Imaging

All fluorescence and DIC images were acquired on an Olympus BX61 microscope using a Q-imaging Camera with Image Pro plus software® (Mediacybernetics).

### SEM Preparation

Specimens were relaxed by storing at 4°C in a mix of MgCl_2_ (7.5%) and seawater (1∶2). A first short fixation (30 sec at 4°C) was done by adding glutaraldehyde in seawater at a final concentration of 3.7%. Then, samples were transferred to a second fixation mix (glutaraldehyde 2.5%, paraformaldehyde 1%, CaCl_2_ 2,5 mM, NaCl 500 mM, sodium cacodylate 50 mM, pH 7.8) for 1 hour at 4°C. After several washes in ice cold washing buffer (CaCl_2_ 2,5 mM, NaCl 500 mM, sodium cacodylate 50 mM, pH 7.8), a post-fixation step was performed in 1% tetraoxyde osmium in washing buffer during 1 hour. Finally, specimens were washed several times in distilled water. Selected animals were dehydrated in a graded series of alcohol then transferred to amylacetate. Following critical-point drying, the specimens were mounted on copper stubs with silver point, coated with gold (330 A layer) in a Polaron sputtering apparatus, and examined on a JEOL JSM 6100 scanning electron microscope (MEB Microanalyse, ITODYS lab, Université Paris 7).

## Results

In this study, expression patterns in the adult body of *Pleurobrachia pileus* are described for 11 genes (*PpiWntA, PpiWntX, PpiWnt6, PpiWnt9, PpiFzA, PpiFzB, PpiDvl1, PpiDvl2, PpiGSK-3β, Ppiβ-cat, PpiTcf*) which are inferred to function in Wnt signalling based on their orthologies with well-known Wnt signalling genes previously characterised in diverse experimental model species. The phylogenetic analyses establishing these orthology relationships are provided in File S1.

We describe here in details the expression patterns of these genes in the areas of the body for which we have good morpho-anatomical knowledge allowing interpretation of the patterns in terms of structures, cell types and sometimes cellular dynamics. These are the oral region, the comb rows, the aboral sensory complex (polar fields and apical organ) and other epithelial neuro-sensory structures, and the tentacle root. Other expression territories (e.g. within the gastro-vascular system: pharynx and/or endodermal canals; in unidentified cell populations of the ectodermal epithelium; in the tentacular apparatus outside from the tentacle root) will not be described here.

### Evidence for Wnt/β-catenin Activity at the Mouth Margin, a Zone of Intense Cell Proliferation

**Figure 2:**
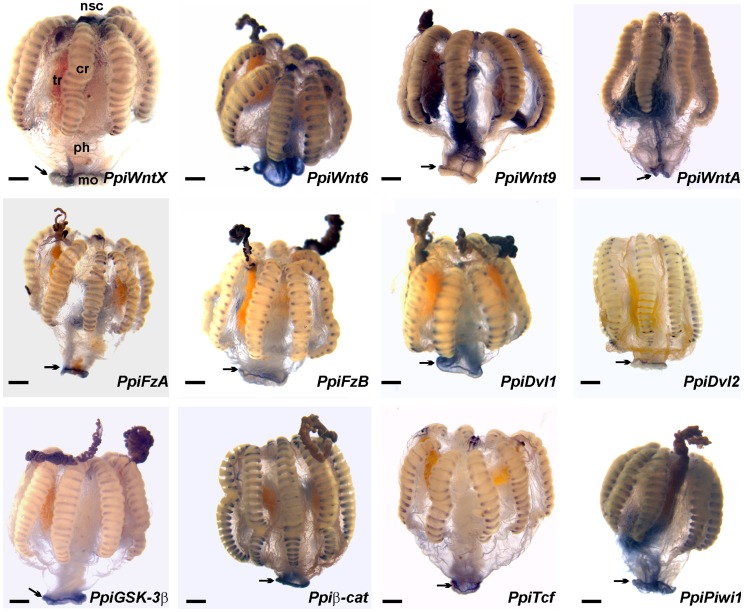
Oral ring of gene expression for Wnt signalling genes and *Piwi* (general views). The pictures show whole adult specimens of *P. pileus* after gene *in situ* hybridisation. Legends in the first picture as in Figure 1; mouth margin indicated by black arrow; oral pole at the bottom in all pictures. Scale bars: 500 µm.

**Figure 3:**
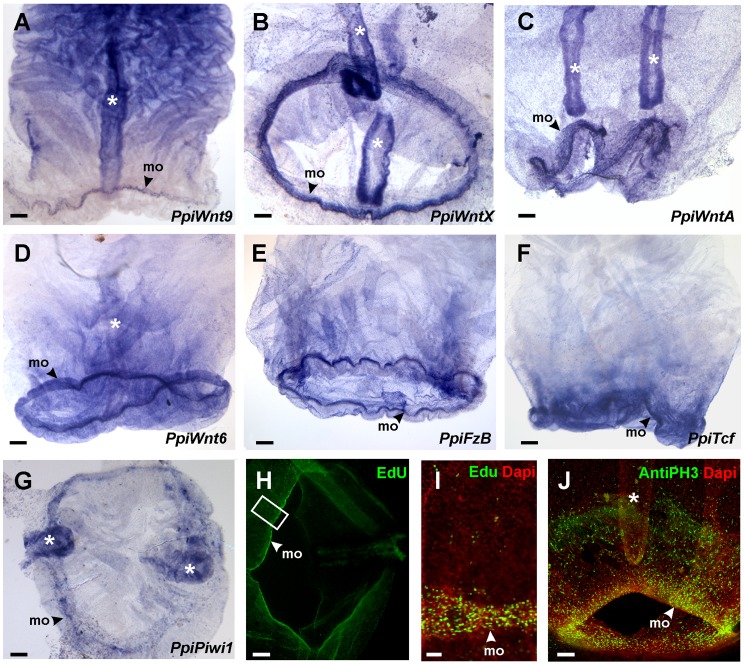
Oral rings of gene expression (details) and evidence for intense cell proliferation around the mouth. The four genes encoding Wnt ligands have graded extensions of their expression domains starting from the mouth margin, with *PpiWnt9* (A) expression restricted to a very thin band, whereas *PpiWntX* (B) and *PpiWntA* (C) are expressed in a distinctly wider belt around the mouth, and *PpiWnt6* transcripts are detected in an even broader domain (D). Details of expression at the oral pole are also shown for two other genes, *PpiFzB* (E), *PpiTcf* (F), and for the stem cell marker gene *PpiPiwi1* (G). Most of these genes are also expressed in the two paragastric canals (white stars in A–D, G, J), elements of the gastro-vascular system that run parallel to the pharynx, from the stomach to the mouth area. The mouth border is a region of intense cell proliferation, as evidenced by EdU DNA label incorporation after a 2 h pulse (H) (I: higher magnification of the area boxed in H) and by immunolabelling of mitotic chromosomes using an anti-phospho-Histone H3 antibody (J). The arrowhead points to the mouth margin (mo) in all pictures. Scale bars: (A–H, J) 100 µm; (I) 10 µm.

All genes investigated in this study were found strongly expressed along the margin of the mouth (black arrows in Fig. 2; arrowheads in Fig. 3A–F; see controls in File S2). Interestingly, the extension of this oral expression domain differs significantly between the four genes encoding Wnt ligands. *PpiWnt9* transcripts are restricted to a very narrow oral stripe and within this stripe staining is associated with a subpopulation of cells, more or less scattered along the mouth margin (Fig. 3A). *PpiWntA* and *PpiWntX* are more homogeneously expressed and in a wider stripe (its width of about 4–5 cellular diameters) (Fig. 3B, C), whereas *PpiWnt6* has an even broader expression domain in the ectodermal epithelium of the oral region, with a maximum level of transcript concentration along the mouth margin (Fig. 2; Fig. 3D). Among the other genes, *PpiFzA, PpiDvl1*, *PpiGSK-3β, Ppiβ-cat* and *PpiTcf* (Fig. 3F) are expressed more or less like *PpiWntA* and *PpiWntX,* while the expression domains of *PpiFzB* (Fig. 2, Fig. 3E) and *PpiDvl2* (Fig. 2) are narrower, but yet more extended than that of *PpiWnt9*.

Localised expression of Wnt/β-cat pathway genes in the oral region prompted us to examine by three different approaches the possibility of the presence in this area of a previously-overlooked stem cell population. Firstly, new ISH experiments using an antisense RNA probe for the stem cell marker gene *PpiPiwi1* [32] revealed localised expression of this gene around the mouth margin (Fig. 2; Fig. 3G). Secondly, nuclei having incorporated the thymidine analogue EdU after 2 h (Fig. 3H, I) or 14 h (not shown) pulses were much more abundant near the mouth margin than in the rest of the oral region. Finally, a considerable concentration of anti-phospho-histone H3 immunoreactive nuclei was detected in the same area (Fig. 3J). These results indicate that the mouth margin is a region of intense cell proliferation and probably houses a population of stem cells.

### Expression of Wnt Signalling Genes Associated with Areas Rich in Undifferentiated Cells in the Tentacle Root

**Figure 4:**
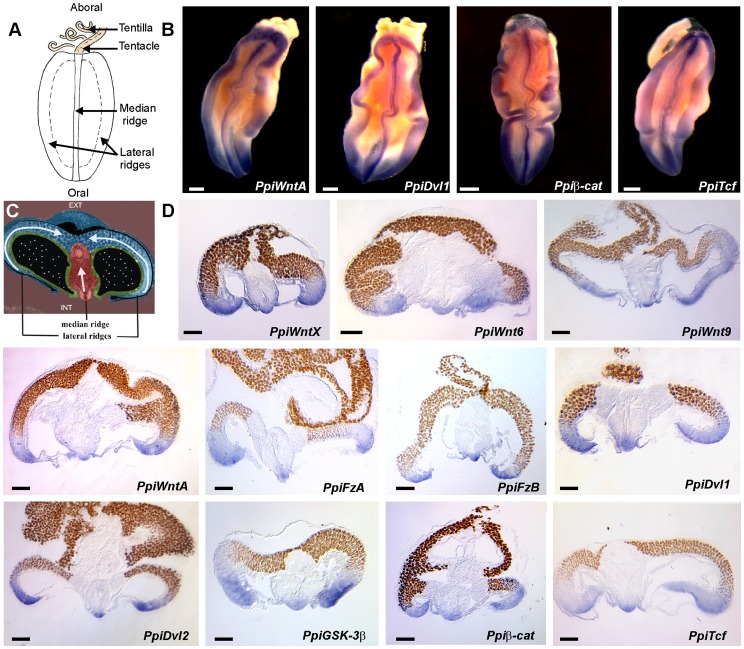
Gene expression in the tentacle root. (A) Schematic representation of the *P. pileus* tentacle root in internal view, notably showing the three characteristic longitudinal ridges containing the stem cells of colloblasts (lateral ridges, [32]) and of muscle cells (median ridge, [34]). (B) Whole-mount *in situ* hybridisation in dissected tentacle roots for four Wnt signalling genes. Orientation as in (A). (C) Schematic view of the tentacle root in transverse section (at about midlenght). The arrows indicate the direction of cell movement and differentiation along the colloblast (lateral light blue arrows) and muscle (median light pink arrow) cellular lineages. Colour code: blue: ectodermal epithelium; green: endodermal epithelium; red: mesogleal component of the tentacle root (muscle and nervous system); purple: body mesoglea; black: lumen of the tentacle sheat; black with white dots: lumen of the tentacular gastro-vascular canals. EXT: external side (i.e. towards body periphery); INT: internal side (i.e. towards pharynx). (D) Gene expression in transverse cryosections of the tentacle root. Orientation as in (C). Scale bars: 100 µm.

The various genes investigated share similar expression characteristics in the tentacle root. They are all strongly expressed on the internal side of the tentacle root in three longitudinal bands, as shown for *PpiWntA, PpiDvl1, Ppiβ-cat* and *PpiTcf* in Fig. 4B: one median band and two marginal bands, corresponding respectively to the median ridge (which houses the stem cells that give rise to the tentacular musculature, [34]) and to the lateral ridges (where stem cells of the colloblasts are concentrated, [32]) (Fig. 4A). Examination of whole mount ISH preparations in transverse cryosections (Fig. 4D) confirms that all genes are overexpressed at the distal extremities of the median and lateral lobes of the tentacle root, and since both behave like “cellular conveyor belts” [45] as described in [32], [34] and summarised in Fig. 4C (arrows), we conclude that the Wnt/β-catenin pathway is activated in the less differentiated stages of the colloblast and muscle cell lineages and turned off as differentiation proceeds.

### Expression of Wnt Signalling Genes within the Aboral Neuro-sensory Complex (Apical Organ and Polar Fields)

**Figure 5:**
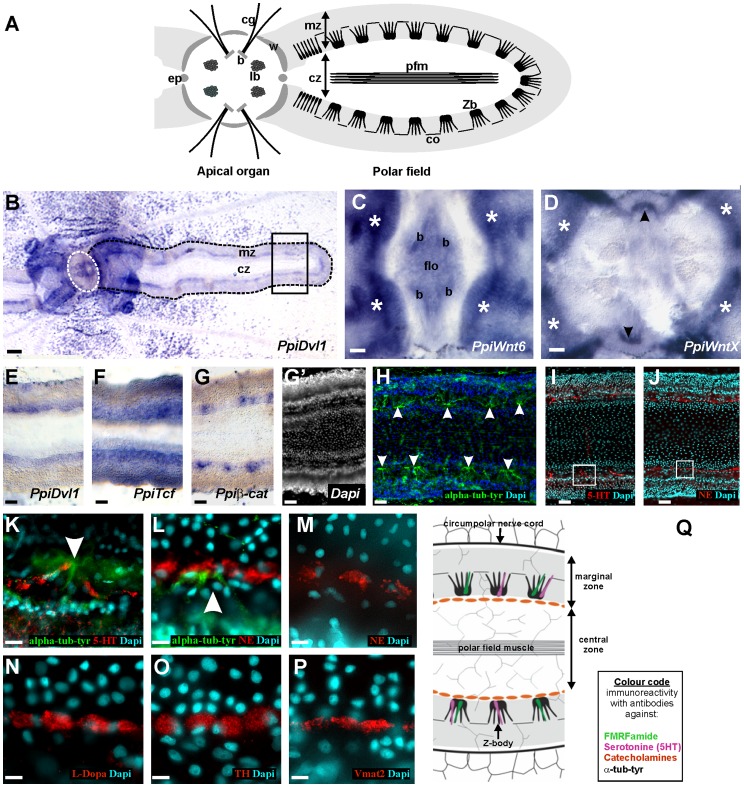
Gene expression in the aboral neuro-sensory complex and architecture of the polar field nervous system. (A) Summary of the morphology of the aboral neuro-sensory complex (polar field represented only on one side). Abbreviations: b: balancers; cg: ciliated groove; co: connection between Z-bodies; cz: central zone; ep: epithelial papilla; lb: lamellate bodies; mz: marginal zone; pfm: polar field muscle; Zb: Z-body. General distribution of transcripts within the aboral neuro-sensory complex is shown for *PpiDvl1* in (B), where the white dotted line delineates the apical organ and the black dotted line represents the polar field outline. The two distinct zones making up the polar field are labelled as mz (marginal zone) and cz (central zone). Detailed views of *in situ* hybridisation in the central area of the aboral neuro-sensory complex are given for *PpiWnt6* (C) and *PpiWntX* (D). Both genes are strongly expressed in the proximal extremities of the polar field marginal zones (asterisks). In addition, within the apical organ, *PpiWnt6* (C) is expressed in the balancers (b), in the epithelial floor (flo), and in the lithocytes (not visible because not on focus), and *PpiWntX* is expressed in two peripheral transverse spots in the tentacular plane (arrowheads in D). In the polar fields outside from their most proximal area, examples are shown in (E–G) (corresponding to the boxed area in B) for each of the three observed categories of gene expression patterns: a continuous band along the inner border of the marginal zone (E); same territory but with additional weaker expression in the rest of the marginal zone (F); or discontinuous spots along the inner border of the marginal zone, this situation being observed only for *Ppiβ-cat* (G). These spots correspond to the anti-tyrosylated-α-tubulin immunoreactive Z-bodies (white arrowheads in H), as shown by comparison of the distribution of cell nuclei in Dapi counter-staining of *Ppiβ-cat in situ* hybridisation (G’) and of anti-tyrosylated-α-tubulin immunostaining (H). The distribution of anti-serotonin (5-HT) immunoreactive neurons in the polar field is shown in (I) and (K), the latter corresponding to a detailed view of the area boxed in (I) with additional anti-tyrosylated-α-tubulin staining. The inner region of the polar field marginal zone furthermore contain a continuous row of catecholaminergic elongated cells, strongly immunoreactive with antibodies directed against norepinephrin (NE) (J, L, M), L-Dopa (N), tyrosine hydroxylase (TH) (O), and Vmat2 (P) (L–P: higher magnification views of area boxed in J). The white arrowhead in (K) and (L) points to a Z-body. The neuro-sensory architecture of the polar field is summarised in (Q). Scale bars: (B) 50 µm; (C–J) 20 µm; (K–P) 10 µm.

With the exception of *PpiWnt9,* all investigated genes were found to be expressed in the aboral sensory complex. The characteristic anatomy of this region is summarised in Fig. 5A. Since there is some redundancy in the expression features of these genes in the apical organ and polar fields, the patterns are detailed in Table 1 and only selected examples are illustrated in Fig. 5. Within the apical organ, there are three sites where transcripts of more than one gene could be detected: the lithocytes (mineralised cells forming the statolith – not shown), the four balancers (agglomerated long cilia which support the lithocyte) and the epithelial floor (outside balancers). Two genes, *PpiWnt6* (Fig. 5C) and *PpiTcf* (not shown) cumulate these three expression territories. Furthermore, an additional expression site, two symmetrical transverse spots at the apical organ periphery in the tentacular plane, was seen only for *PpiWntX* (arrowheads in Fig. 5D). This site does not correspond to any previously described structure of the apical organ anatomy.

**Table 1:** Summary of gene expression patterns in the aboral neuro-sensory complex.

	Expression in the apical organ				Expression in the polar fields		Expression in theciliated grooves
	Litho-cytes	Balancers	Epithelial floor	Two transverse spots,near border,ontentacular plane	Intense staining of themarginal zone near theapical organ (proximal/distal gradient)	Elsewhere in themarginal zone:	
*PpiWnt9*	**–**	**–**	**–**	**–**	**–**	**–**	**–**
*PpiWnt6*	**+**	**+**	**+**	**–**	**+**	Continuous band along inner border	faint
*PpiWntA*	**–**	**–**	**–**	**–**	**+**	Continuous band along inner border	faint
*PpiWntX*	**–**	**–**	**–**	**+**	**+**	Continuous band along inner border	very strong
*PpiFzA*	**+**	**–**	**–**	**–**	**+**	Continuous band along inner border	rather strong
*PpiFzB*	**+**	**–**	**–**	**–**	**+**	Throughout marginal zone; highest along inner border	rather strong
*PpiDvl1*	**–**	**+**	**–**	**–**	**+**	Continuous band along inner border	faint
*PpiDvl2*	**+**	**–**	**–**	**–**	**+**	Throughout marginal zone; highest along inner border	faint
*PpiGSK-3β*	**+**	**–**	**–**	**–**	**+**	Throughout marginal zone; highest along inner border	rather strong
*Ppiβ-cat*	**+**	**–**	**–**	**–**	**+**	Expression restricted to Z-bodies	**–**
*PpiTcf*	**+**	**+**	**+**	**–**	**+**	Throughout marginal zone; highest along inner border	rather strong

All genes (except *PpiWnt9*) are strongly expressed in the proximal region of the polar fields (i.e. close to the apical organ) (Fig. 5B, C, D). For these genes, staining intensity reaches a maximum at the four places where the polar field marginal zone contacts the apical organ (asterisks in Fig. 5C, 5D), coinciding with where pools of putative stem cells have been previously identified [32]. Original results of EdU labelling experiments shown in File S3 confirm that the proximal polar field region is an area of intense cell renewal.

All genes except *PpiWnt9* are also expressed in the differentiated part of the polar fields (i.e. far from the apical organ), namely within a thin band positioned along the inner border of the polar field marginal zone (Fig. 5E), in some cases with staining also visible elsewhere in the marginal zone (Fig. 5F), and in the case of *Ppiβ-cat* only, in distinct and well-separated spots (Fig. 5G). This latter pattern is particularly interesting because these spots closely match recently described [Jager et al. 2008, 2011] neural structures called the Z-bodies, resembling small ganglia and aligned along the inner edge of the marginal zone. They are visualised by their strong immunoreactivity in anti-tyrosylated-α-tubulin stained preparations (arrowheads in Fig. 5H), and their precise location can be easily determined on ISH preparations of polar fields by looking at DAPI counter-staining (Fig. 5G’), thanks to the very distinctive distribution of cell nuclei within and around Z-bodies (see in Fig. 5H).

The localised expression of several Wnt/β-catenin pathway genes in the inner part of the polar field marginal zone thus correlates with remarkable local differentiation of the nervous system in this area. It was previously shown that the Z-bodies contain a subpopulation of anti-FMRF-amide immunoreactive cells [46]. We report here two additional observations confirming that the inner portion of the polar field marginal zone contains a very distinctive nervous system. Using an antibody against serotonin (5HT), we identified a specific subpopulation of neurons within the Z-bodies (Fig. 5I, 5K). Using several antibodies directed against catecholamines and associated proteins, strong staining was obtained in a continuous row of large cells, parallel to the Z-body alignment but in a slightly more internal position (Fig. 5J, 5L–5P). These cells do not have a neuronal morphology, yet their catecholaminergic nature is strongly supported by immunoreactivity not only with antibodies against norepinephrin (Fig. 5J, 5L, 5M) and L-dopa (Fig. 5N), but also with anti-TH (tyrosine hydroxylase enzyme) and anti-Vmat2 (catecholamine transporter) antibodies (Fig. 5O and 5P). Our current understanding of the architecture of the nervous system of the polar field margin zone is summarised in Fig. 5Q.

### Expression of Wnt Signalling Genes in other Neuro-sensory Structures

**Figure 6:**
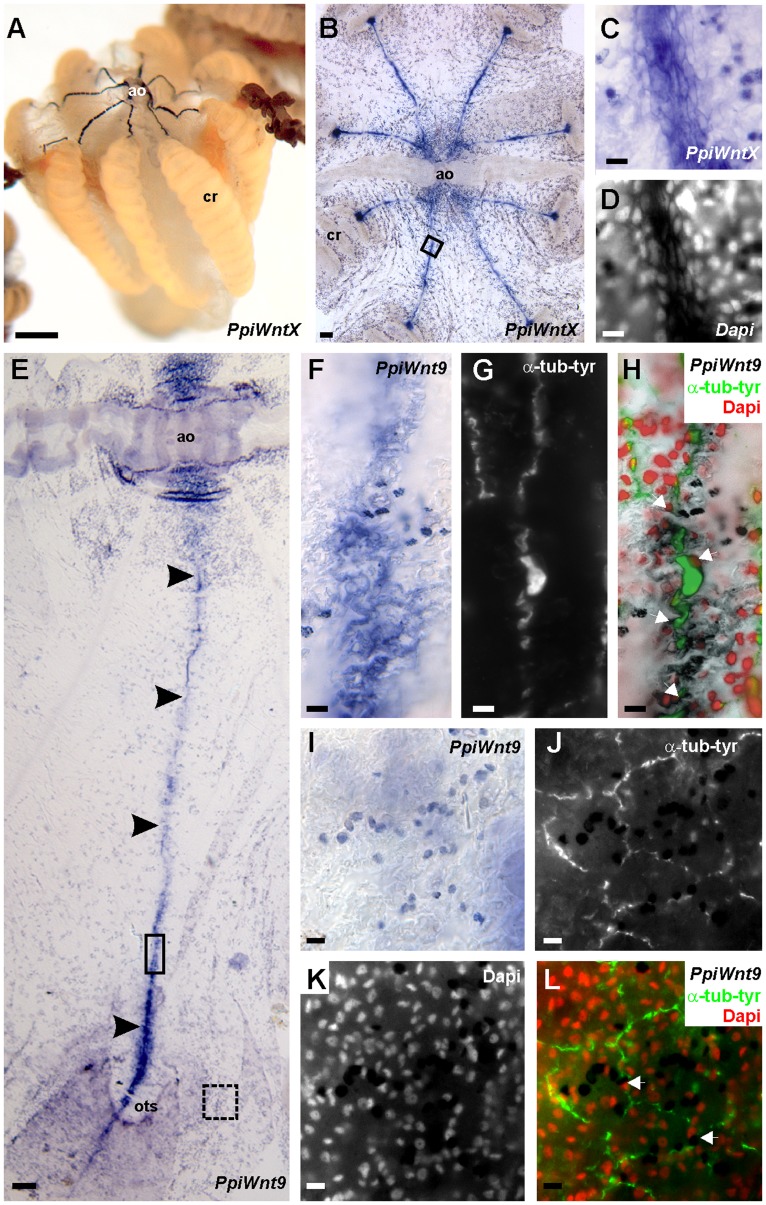
Gene expression in the ciliated grooves and in the juxta-tentacular nerve cord. The *PpiWntX* gene is strongly expressed in the eight ciliated grooves as shown in general views with incident light (A) and transmitted light (B) (ao: apical organ; cr: comb row). Note that for this specimen, duration of the revelation step was short explaining why no staining is seen in the aboral neuro-sensory complex. *PpiWntX* transcripts localise to the elongated cells that make up the ciliated grooves (C, D: detailed views corresponding to the area boxed in B). The *PpiWnt9* gene is expressed in the condensation of the polygonal nerve plexus called the juxta-tentacular nerve cord (arrowheads in E) from the apical organ (ao) to the opening of the tentacle sheath (ots). Within this structure, *PpiWnt9* transcripts are concentrated in the cell bodies of neurons (white arrows in H) as shown by comparison at high magnification of *in situ* hybridisation (F) and anti-tyrosylated-α-tubulin counterstaining (G, H). *PpiWnt9* expression in scattered epidermal cells (I) also associates with neural cell bodies of the polygonal nerve net (anti-tyrosylated-α-tubulin counterstaining in J; Dapi counter-staining in K) as shown by distribution of the *PpiWnt9* staining in merged view (white arrows in L). (F, G, H) are higher magnification views of the region boxed in full line in (E); (I–L) are high magnification pictures of the area boxed with dotted line in (E). Scale bars: (A) 500 µm; (B, E) 50 µm; (C, D, F–L) 10 µm.

Outside the aboral neuro-sensory complex, several genes are expressed in the eight ciliated grooves, the specialised epithelial structures that connect the apical organ to each of the eight comb rows. Here staining was particularly intense for *PpiWntX* (Fig. 6A to 6D), but was also observed for all other genes except *PpiWnt9* and *Ppiβ-cat* (Table 1).

The *PpiWnt9* gene (which is not expressed in the aboral sensory complex or the ciliated grooves) is strongly expressed in a longitudinal condensation of the epithelial nerve plexus called the juxta-tentacular nerve cord [46] (Fig. 6E to 6H), as well as throughout the epithelial nerve plexus (Fig. 6I to 6L). In both situations, counter-staining with the anti-tyrosylated-α-tubulin antibody, a pan-neural marker in ctenophore [46] confirms localisation of the signal in nerve cells (Fig. 6H, 6L). Of note is that *PpiWnt9* expression in the juxtatentacular nerve cord was seen only in its portion spanning from the apical organ to the tentacular sheath opening (not in the portion of this nerve cord located on the oral side of this opening). Expression in the juxta-tentacular nerve cord could not be detected for any of the other 10 genes investigated.

### Insights into Cellular Dynamics within Mature Combs from Expression of Ciliogenesis Markers

Prior to description of gene expression patterns in the comb rows, it will be useful to provide some background about the organisation of the cellular component of combs (basal cushions) and to give some insights about cellular dynamics within them based on original expression patterns of some ciliogenesis genes.

**Figure 7:**
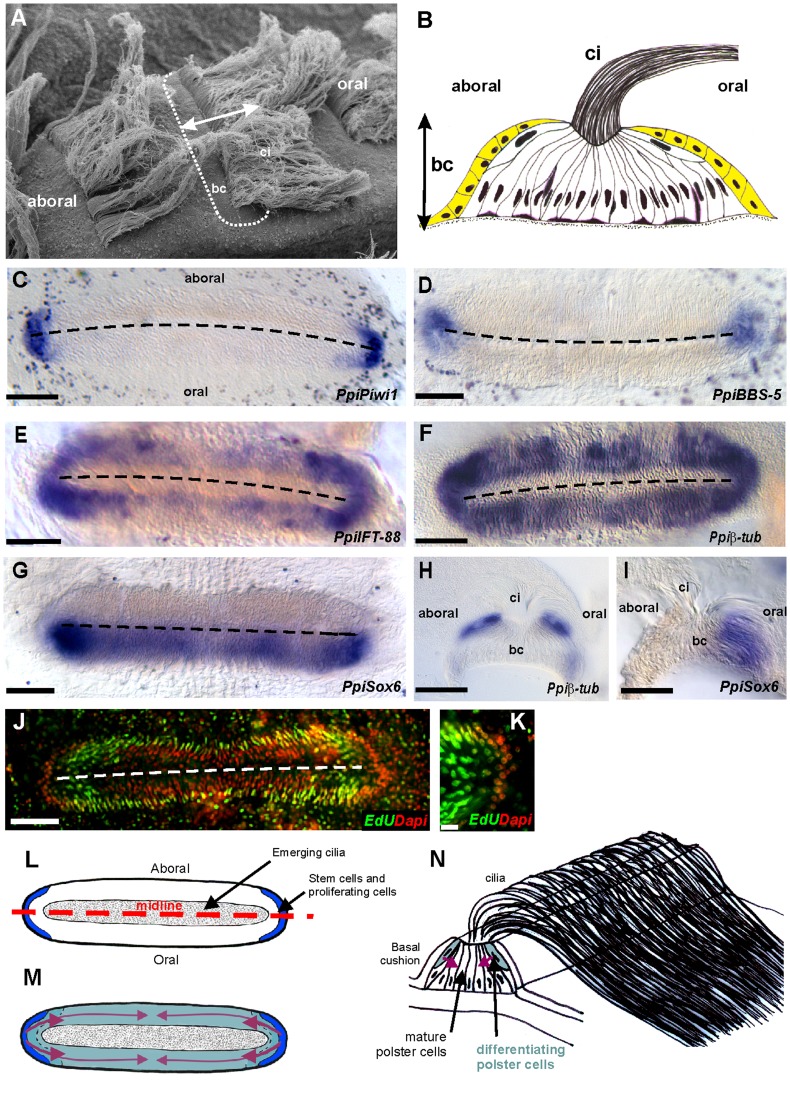
Organisation and cellular dynamics of the comb. (A) Scanning electron microscopy view of two successive combs along a comb row. The dotted line materialises the outline of the comb basal cushion (bc), the cilia (ci) emerging along its midline. (B) Schematic transverse section of a basal cushion according to the double-arrowed line in (A) (after [70]). Polster cell bodies are represented in white, the covering epithelium in yellow, and nerve cells in purple (bc: basal cushion; ci: cilia). (C) Expression in a basal cushion of the stem cell marker *PpiPiwi1*. (D) Expression of the early ciliogenesis marker *PpiBBS-5*. (E) Expression of the later ciliogenesis marker *PpiIFT-88*. (F) Expression of *β-tubulin*. (G) Expression of *PpiSox6*, a marker of mature polster cells of the basal cushion oral half. Transverse sections of the basal cushion after *in situ* hybridisation are shown for *Ppiβ-tub* in (H) and for *PpiSox6* in (I); bc: basal cushion; ci: cilia; see (B) for interpretation of these pictures. (J) Distribution of EdU labelled nuclei after a pulse of 20 h and a chase of 5 days, showing that the youngest polster cells are those located near both comb extremities as well as those situated on the border all around the basal cushion. The detailed view in (K) shows that in addition to differentiated nuclei, some undifferentiated nuclei concentrated at the extremity have retained the label (putative stem cells; see [32]). (L–N) Model of cellular dynamics within the basal cushion derived from these data. Stem cells and proliferating progenitors are segregated at both extremities (L) then differentiating polster cells travel (purple arrows in M, N) below the covering epidermis to reach their final destination either close to the extremity (growth in length) or all around the border (growth in width). In (C–G) and (J) the dotted line materialises the basal cushion midline and the aboral pole is on top. Scale bars: 100 µm in all pictures except (K) (10 µm).

The structure of the comb basal cushion, illustrated in external morphology in Fig. 7A and in transverse section in Fig. 7B, is morphologically rather simple. The elongated cell bodies of the ciliated cells (polster cells) are densely packed, their orientation being perpendicular to the axis of main length of the comb. Due to this arrangement of the polster cell bodies, the comb basal cushion has a characteristic striated aspect in tangential views using DIC light microscopy (e.g. Fig. 7D,F). All poster cells have their apical pole close to the comb midline, which is therefore the insertion line of the swimming paddle, made of the co-apted cilia of all poster cells housed in a given basal cushion (Fig. 7A). In transverse section, the polster cell bodies display a fan-like arrangement, and the whole structure is covered by an epithelium interrupted only along the midline where the cilia emerge (Fig. 7B). Transmission electron microscopy studies have reported the presence of nerve cells in this epithelium and in between the polster cell bodies [51].

In a previous study, two populations of putative stem cells each localised at one extremity of the comb basal cushion have been identified, suggesting that comb growth in length and/or cell renewal in the basal cushion involves undifferentiated progenitors coming from the tips [32]. In consistence with this hypothesis, the *P. pileus* orthologue of the early ciliogenesis marker *BBS-5* is expressed at both extremities of the basal cushion (Fig. 7D), in a slightly broader domain than the stem cell marker *PpiPiwi1* (Fig. 7C). The late ciliogenesis markers *IFT-88* and *β-tubulin* have much broader expression domains, the latter extending over the whole surface of the basal cushion apart from a conspicuous interruption along the midline (Fig. 7E, 7F). Examination of transverse sections of combs stained with β-tubulin antisense RNA reveals absence of staining in the differentiated polster cell bodies, but strong staining in a superficial position just below the covering epithelium, in elongated cells with a large nucleus (Fig. 7H). This is consistent with the observed interruption of β-tubulin expression along the midline in tangential view (Fig. 7F), as the covering epithelium does not reach the midline (Fig. 7B). These data, together with results of EdU incorporation assays ([32] and this study Fig. 7J, 7K) suggest that ciliated cell progenitors occur not only towards the comb extremities, but also in a superficial position above the differentiated polster cells throughout the basal cushion except along midline. Our proposed model of cellular dynamics within the basal cushion is illustrated in Fig. 7L to 7N.

Of interest to the following is that genes expressed in polster cell progenitors (or in differentiating polster cells) have an expression domain that does not extend to the midline area, with a sharp limit corresponding to the edge of the covering epithelium (as visible in Fig. 7E, 7F), whereas the expression domain of genes expressed in differentiated polster cells reaches the midline (shown in Fig. 7G and 7I for *PpiSox6*, a marker of mature polster cells of the oral basal cushion half, [52]), owing to the fan-like arrangement of these cells.

### Expression of Wnt Signalling Genes within the Comb Rows

**Figure 8:**
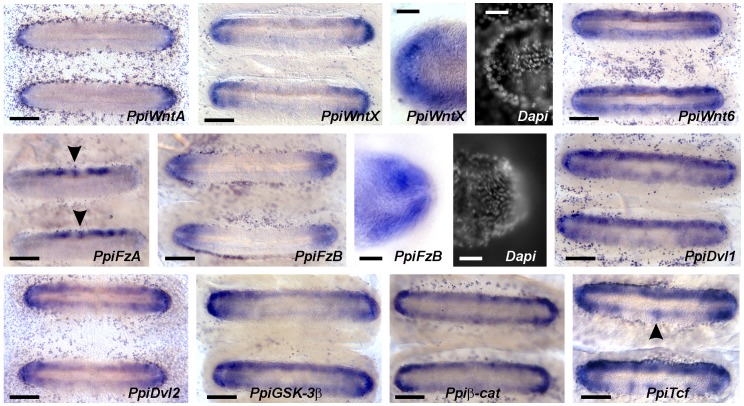
Expression of Wnt signalling genes in comb rows. Two successive basal cushions are shown for each gene, with details of the comb extremity and Dapi counterstaining provided for *PpiWntX* and *PpiFzB*. The arrowhead for *PpiFzA* points to the central aboral spot of expression (see text); arrowhead for *PpiTcf* indicates central oral spot of expression (see text). Aboral pole on top in all pictures. See also pictures of gene expression at lower magnification in ten successive combs in File S4. Scale bars: 100 µm, except for details of *PpiWntX* and *PpiFzB* expression (20 µm).

All investigated genes except *PpiWnt9* are expressed in the basal cushions of the combs (Fig. 8 and File S4). Three genes are strongly expressed only towards both extremities of the basal cushion: two of the four *P. pileus* genes encoding Wnt ligands, *PpiWntA* and *PpiWntX*, and one of the two genes encoding Wnt receptors, *PpiFzB*. The narrow crescent-shaped expression domain of *PpiWntA* reminds that of stem cell markers (Fig. 7C; [32]) whereas the expression domains of *PpiWntX* and *PpiFzB* are broader. In addition, an original feature of *PpiFzB* expression is the presence of an unstained circular area nested within the expression domain (see detail in Fig. 8). This area of low transcript concentration is located on the comb midline just before the apex of the basal cushion. This hole does not match the previously-identified stem cell population, which forms a terminal crescent, and neither corresponds to any known morphological structure.

Among the two other genes encoding Wnt ligands, *PpiWnt9* is not expressed in the basal cushion (see File S4); on the contrary, *PpiWnt6* is expressed throughout the basal cushion except in an elongated area along midline (Fig. 8), a pattern highly reminiscent of that of β-tubulin (Fig. 7F). Remarkably, the three Wnt genes expressed in the basal cushion have nested expression domains starting from the comb extremities, *PpiWntA* having the most restricted expression zone and *PpiWnt6* the broadest.

All other investigated Wnt signalling genes show some degree of oral/aboral polarity of transcript distribution in the comb basal cushion, with expression being in all cases higher in the aboral half. The most sharply polarised expression pattern is that of the Wnt receptor *PpiFzA*. Transcripts of this gene are restricted to a rather narrow band along the aboral edge of each comb, and they are excluded from both the midline area and the extremities. Within this band *PpiFzA* expression appears patchy, notably with one particularly conspicuous spot consistently observed at the centre of the basal cushion aboral half (arrowheads for *PpiFzA* in Fig. 8; see also File S4).

Oral/aboral polarity within the basal cushion also characterises the expression of the investigated components of the transduction cascade. Both *Dvl* paralogues, *GSK-3β, β-cat* and *TCF* are expressed throughout the surface of the basal cushion except in the midline area, but at much higher level at both extremities, and with slight oral/aboral polarity (expression higher in the aboral half). In the oral half, a small central spot of higher transcript concentration is observed at least in some combs/individuals for *PpiGSK-3β, Ppiβ-cat* and *PpiTcf* (see arrowhead for the latter gene in Fig. 8). The expression pattern of *PpiTcf* otherwise stands out by its more patchy aspect. None of the Wnt signalling genes expressed in the basal cushion show any signal in the midline area, implying that their expression is turned off as polster cells mature.

## Discussion

### An Oral Wnt Centre in the Adult Ctenophore

It has become apparent in recent years that in phylogenetically distant animal lineages the setting up, maintenance and patterning of the primary body axis involves a gradient of Wnt/β-catenin activity with a maximum at one pole. The Wnt centre is posterior in bilaterian embryos [12], [13], [53], oral/posterior in cnidarian embryos, larvae and adult polyps [4], [18], [19], [21], [23], [28], [54], and posterior in a sponge larva [7], [37], [55].

However in the ctenophore *Mnemiopsis leidyi* [8] most Wnt signalling genes (including the four Wnt ligands) were rather surprisingly found to be expressed only after the establishment of a morphologically defined oral/aboral axis. Furthermore drugs that interfere with the activity of the pathway did not appear to affect axis formation [8]. At a later stage (cydippid larva), Wnt ligands and most other components of the pathway were expressed in multiple discrete regions, in some cases with good correspondence with the expression data reported here for orthologous genes in the adult *Pleurobrachia pileus* (e.g. *WntX* strongly expressed in the ciliated grooves; most genes expressed in the tentacle roots, etc.) and for some genes on the contrary with marked discrepancy (e.g. *β-cat* in *M. leidyi* cydippid expressed everywhere except in the cells forming the comb plates), but with no evidence for an oral Wnt centre.Pang *et al.* [8] concluded from their data that in ctenophore the Wnts probably do not play a role in early axis specification.

Our expression analyses demonstrate that unlike in *M. leidyi* embryos and larvae, there is an oral Wnt centre in the adult of the ctenophore *P. pileus* (Fig. 2, 3), with strong expression around the mouth margin of the four Wnt ligands as well as both Frizzled receptors and components of the β-catenin pathway intracellular machinery (*Dvl, GSK-3β, β-cat, TCF*). Furthermore, Wnt ligands have nested expression domains starting from the oral pole, a situation somewhat reminiscent of nested Wnt expression in cnidarian planula larvae [4], [19], [23].

How to reconcile these apparently contradictory observations in *P. pileus* and *M. leidyi*? A first scenario would invoke loss of the oral Wnt centre only in the lineage of *M. leidyi* (which unlike *P. pileus* belongs to the ctenophore order Lobata). It is not possible at the moment to exclude this hypothesis, in the absence of Wnt expression data in the adult of *M. leidyi* as well as during embryogenesis in *P. pileus*. However, a more likely explanation in our view would be that in ctenophores, Wnt/β-catenin signalling has lost its early role in establishment of the oral/aboral axis during embryogenesis, but is nevertheless still involved in its maintenance/patterning in the adult. Consistent with this idea, ctenophore embryogenesis stands apart among non-bilaterians by its strongly mosaic nature and highly stereotyped cleavage pattern [56], in contrast to the much more regulative development of sponges and cnidarians. In terms of developmental mechanisms, acquisition of such a mosaic mode of embryogenesis (clearly apomorphic in ctenophores) implies that cell fate has become less dependent upon communication between cells. A parallel example among bilaterians is the complete loss of Wnt function in setting up the antero-posterior axis in flies, a derived lineage of insects in which early development does not rely anymore on cell-cell communications [2], [13]. On the contrary, regulative processes are prevalent in the adult body of ctenophores, as indicated by their high regenerative capacities [57], and by intense cell turnover taking place continuously in all regions of the body [32], [34]. Positional cues are thus probably required to maintain tissue homeostasis and body shape, as it is the case in adult cnidarians [26]. Functional experiments will be required to determine if the oral Wnt-expressing pole of adult ctenophores behaves like an organiser.

### Ancient Role of the Wnt/β-catenin Pathway in Undifferentiated Proliferating Cells

The observed correlation, throughout the ctenophore body, between strong expression levels of Wnt signalling genes (including those coding for actors of the β-catenin pathway) and areas of stem cell concentration and intense cellular division suggests an ancient role of the Wnt/β-catenin pathway in promoting cell proliferation and possibly other properties of stem cells such as self-renewal and potency. This correlation was observed in the three body areas with localised stem cell populations and spatially ordered cell renewal (combs, tentacle root, polar fields). Likewise, the circular area around the mouth where all investigated genes are strongly expressed is also characterised by intense cell proliferation and strong *PpiPiwi1* expression (Fig. 3G–3J). Of note is that a role of Wnt/β-catenin signalling in regulating cell proliferation at the oral pole would not be contradictory with the above suggestion that the oral Wnt centre might control oral/aboral polarity like in the other main animal lineages. Indeed, in bilaterians, the posterior Wnt organiser controls both antero-posterior polarity and posterior growth [53].

Studies in mammalian models have pinpointed the central role of the Wnt/β-catenin signalling pathway in stem cell biology [9], [58]. In many cases, Wnt signalling promotes cell proliferation and stem cell renewal [9], [59]–[62], but in bilaterians there are also examples in which Wnt/β-catenin signalling on the contrary acts to inhibit cell proliferation and to promote cell differentiation, like in the neural ventral midline of the annelid *Platynereis dumerilii* [63] or in mammalian skin-derived interfollicular progenitor cells [64]. Of these two situations, association of an activated state of the Wnt/β-catenin pathway with stemness and cell proliferation is probably ancestral at the level of Eumetazoa, as suggested by the results described here for the ctenophore *P. pileus*. This also finds support from experimental evidence in *hydra*, since *TCF* was found to be among the few conserved genes strongly expressed in stem cells of all three cellular lineages in this cnidarian polyp [65]. However, even in non bilaterians the picture is in fact not so simple since on the other hand the Wnt β-catenin pathway has been shown to induce differentiation of interstitial stem cells into a particular type of nematocytes in hydra [66], and in *P. pileus* (this study) there is at least one instance where the pathway seems activated in a differentiated context (strong β-cat expression associated with the Z-bodies in the polar fields, Fig. 5G). Future progress in our understanding of the early evolution of Wnt functions with respect to stem cells can be expected to come out from in depth studies of cellular and molecular mechanisms in established experimental models, but also and importantly from comparative expression studies of Wnt signalling genes in a variety of taxa, notably cnidarian and sponge species.

### Wnt Signalling and Local Polarities within the Ctenophore Comb Rows

The highly structured spatial distribution of transcripts of multiple Wnt signalling genes observed in *P. pileus* within the basal cushions indicates that co-option of this molecular machinery contributed to the emergence of comb rows (an evolutionary novelty) during evolution along the ctenophore lineage. The distinctive expression patterns of the two Frizzled receptors in the basal cushion furthermore suggest that Wnt signalling regulates polarity within this structure according to two perpendicular directions, even though gradients of Wnt ligands are likely oriented only along the comb midline. Thus *PpiWntA*, *PpiWntX* and *PpiWnt6* are expressed symmetrically with respect to the midline with staggered expression domains starting from the basal cushion extremity, *PpiWntA* having the most restricted expression zone and *PpiWnt6* the broadest. One of the receptors (*PpiFzB*) is strongly expressed at both basal cushion extremities, symmetrically with respect to the midline, and thus probably provides target cells with information about their position along the midline, certainly a critical parameter notably for regulating cell proliferation vs. differentiation. In contrast, the other receptor *PpiFzA* is expressed only on the aboral side of the midline.

Whatever the exact nature of these *PpiFzA*-expressing cells, the sharp oral/aboral polarity of *PpiFzA* expression (together with polarised expression of *PpiGSK-3β, Ppiβ-cat* and *PpiTcf*) is quite remarkable since morphologically the basal cushion is essentially symmetric with respect to the midline, notably with polster cell bodies of the oral and aboral halves being identical from a cytological viewpoint (Fig. 7B). It was already known, however, that oral vs. aboral polster cells have distinct molecular identities (the first express *PpiSox6*, not expressed in the latter, [52]). Asymmetry of the comb with respect to the midline is certainly important for mechanical reasons since beating power strokes during normal swimming (with mouth forwards) are directed towards the aboral pole [67]. Therefore it can be postulated that the cilia of oral vs. aboral polster cells have different structural and functional properties. This aboral/oral polarity of the basal cushion being a clear case of planar cell polarity, the very distinctive expression pattern of *PpiFzA* (compared to other investigated genes) might reflect an involvement here of the Fz-PCP pathway [11]. A last interesting observation is that the expression pattern of *PpiFzA* in fact integrates positional information along both spatial coordinates, since in addition to oral/aboral polarity, it also displays polarity between extremity and centre, with no expression at the latter pole, and a distinct circular spot of expression positioned at the centre (Fig. 8). This also holds true, albeit in a less spectacular way, for components of the intracellular Wnt β-catenin machinery (*Dvl1*, *Dvl2*, *GSK*-3β, *β-cat* and *TCF*).

### Wnts and the Neuro-sensory System

In bilaterians, Wnt signalling not only plays crucial roles during neural development [3], [68], but recently accumulated experimental evidence in mammals have furthermore uncovered essential functions of Wnt components within the mature nervous system [10]. In addition to the control of nerve cell renewal in the adult brain, regulation of synaptic plasticity, and dendrite spine morphogenesis, these functions include involvement of Wnt ligands and receptors in basic neurophysiological processes. For example, Wnts stimulate recycling and exocytosis of neurotransmitter vesicles, control neurotransmitter receptor trafficking and turnover, modulate postsynaptic complex assembly, and trigger effects on NMDA receptors affecting synaptic currents (reviewed in [10]). These roles of Wnt in neuronal activity seem most often performed through β-catenin independent signalling by the Wnt/Ca^2+^ pathway, while in neural development and synapse formation the Wnts act predominantly through the Wnt/β-catenin pathway [10].

At least some of these roles of the Wnts in the neural context are likely to have an ancient evolutionary history, as suggested by expression of Wnt signalling genes in various neuro-sensory structures throughout the adult ctenophore body. Among them, a first category encompasses the three structures that are successively used for propagation of the beating waves born in the apical organ: the four balancers (signal observed for *PpiWnt6, PpiDvl1, PpiTcf*); the eight ciliated grooves (particularly conspicuous staining obtained for *PpiWntX*, Fig. 6A–6B, and weaker expression observed for several other genes, Table 1); and the comb plates (see above). It is indeed a peculiarity of ctenophores that rapid communication between cells to modulate effectors (such as muscle cells or the swimming paddles) can be either by classic transmission of electrical impulses through chemical synapses between neurons (or between a neuron and an effector cell), or by one-to-one mechanical excitation of neighbouring ciliated cells along specialised tracts of epidermis. Beating waves travel from the apical organ through the ciliated grooves and along the comb rows through the latter mechanism and not by neuronal conduction [67], [69]. Thus these structures which exhibit strong Wnt/β-catenin activity, even if of clear mechanosensory nature, cannot be considered truly neural.

Polar fields, another notable site of Wnt/β-catenin activity, were for a long time considered simply as two elongated areas of ciliated epithelium with probable sensory functions. Recently, their importance within the aboral complex as probable areas of neuro-sensory integration has been suggested by new anatomical information obtained through immunofluoresence analyses [46]. These data and additional observations reported here (presence of serotonin-immunoreactive cells interspersed within the Z-bodies, and a continuous row of non-neural catecholaminergic cells adjacent to the Z-body alignment) reveal that polar fields harbour a spectacular and highly organised concentration of distinctive neuro-sensory elements along the internal edge of their marginal zone (summarised in Fig. 5Q). This is exactly where most Wnt signalling genes were found to be expressed at high level, for most of them in the form of a continuous band along the inner border of the marginal zone (Fig. 5E), and for β-cat, only in the Z-bodies (Fig. 5G). Wnt signalling is therefore certainly important in this specialised neural territory and probably works here through the β-catenin pathway.

The expression features of *PpiWnt9* strikingly diverge from that of the other three Wnt ligands. Besides the mouth margin and tentacle root, ectodermal *PpiWnt9* expression was detected only in neurons of the epithelial polygonal nerve plexus, including in its conspicuous condensation called the juxtatentacular nerve cord (Fig. 6E). However, expression of components of the intracellular Wnt/β-catenin machinery was not seen in these *PpiWnt9-*expressing neural structures.

## Conclusions

The expression features, within the adult body of a species belonging to the early-diverging animal lineage Ctenophora, of 11 Wnt signalling genes might be consistent with an ancient origin during animal evolution (i.e. at least dating back to a common ancestor of Eumetazoa) of several key roles that this pathway endorses in “higher” animals (e.g. vertebrates) in development and tissue homeostasis: control of the main body axis through a posterior (in bilaterians)/oral (in coelenterates) signalling centre, control of morphological polarities at lower spatial scales, promotion of cell proliferation and undifferentiated state, and specific additional functions within the neuro-sensory system.

The present work provides a solid platform for future experimental studies targeted at clarifying the functions of Wnt signalling in various contexts both during development and in the adult body in ctenophores. Notably, it will be important to determine whether the Wnt oral centre uncovered in the adult *P. pileus* really plays a role with respect to global oral/aboral body polarity. Indeed an alternative possibility could be that Wnt/β-catenin signalling around the mouth margin would be involved only in local regulations (e.g. of cell proliferation and/or differentiation in this area). Use of pharmacological drugs to block or activate the pathway, and/or gene interference using RNAi to selectively inhibit the activity of genes involved in Wnt signalling, can also be expected to provide insights about functions of the pathway in the control of cell proliferation notably along cellular lineages of the tentacle root conveyor belts, in building specific neuro-sensory structures, and in regulating polarity and regular repetition of the basal cushions within the comb rows.

A further stimulating perspective will be to investigate genes associated with Wnt/Fz signalling through β-catenin independent mechanisms, such as the Fz-PCP (planar cell polarity) and Fz-Rho pathways [11]. The basal cushion represents a particularly promising system to address PCP in ctenophores, because it is a specialised portion of epithelium displaying marked polarity reflected at the level of individual cells as well as of the whole structure, and because its formation can be followed experimentally both under normal conditions (addition of new combs at both extremities of comb rows, [32]) and under regeneration after ablation [57].

## Supporting Information

File S1 **Gene phylogenetic analyses.**
(PDF)

File S2 **Negative (sense probe) and positive (** ***PpiTbxE*** **and** ***PpiPax1*** **) controls for whole mount** ***in situ*** **hybridisation.**
(PDF)

File S3 **DNA label incorporation experiments providing insights into cellular dynamics in the polar fields.**
(PDF)

File S4 **Gene expression in the comb rows.**
(PDF)
