# Supplementary material for: Evidence for Involvement of Wnt Signalling in Body Polarities, Cell Proliferation, and the Neuro-Sensory System in an Adult Ctenophore
Source: PLoS One. 2013 Dec 31;8(12):e84363. doi: 10.1371/journal.pone.0084363 (PMC3877318; doi:10.1371/journal.pone.0084363)
Supplement: File S1 — Gene phylogenetic analyses. (PDF) [file pone.0084363.s001.pdf]

Evidence for involvement of Wnt signalling in body polarities, cell proliferation, and the neuro-sensory system in an adult ctenophore

Muriel Jager, Cyrielle Dayraud, Antoine Mialot, Eric Quéinnec, Hervé le Guyader and Michaël Manuel

Supporting Information File S1: Gene phylogenetic analyses

Content

**p. 2**	**Methods**
	**Abbreviations and colour code used in the trees**
**p. 3**	**Wnt tree**
**p. 4**	**Fz tree**
**p. 5**	**Dvl tree**
**p. 6**	**GSK-3β tree**
**p. 7**	**β-catenin tree**
**p. 8**	**TCF tree**

Methods

Alignments were generated automatically using MUSCLE then checked for manual correction of obvious errors. Ambiguously-aligned zones were removed prior to analyses. Maximum- Likelihood (ML) analyses were performed using the PhyML program (Guindon & Gascuel, 2003), with the LG model of amino-acid substitution and a BioNJ tree as the input tree. A gamma distribution with four discrete categories was selected. The gamma shape parameter and the proportion of invariant sites were optimised during the searches. The statistical significance of the nodes was assessed by bootstrapping (200 replicates).

Abbreviations for taxon names used in the trees

Aqu : *Amphimedon queenslandica* ; Bra : *Branchiostoma floridae* ; Cel : *Caenorhabditis elegans* ; Dme : *Drosophila melanogaster* ; Hsa : *Homo sapiens* ; Hma : *Hydra magnipapillata* ; Mle : *Mnemiopsis leidyi* ; Nve : *Nematostella vectensis* ; Ppi : *Pleurobrachia pileus* ; Spu : *Strongylocentrus purpuratus* ; Tad : *Trichoplax adhaerens*.

Colour code

Genes from *Pleurobrachia pileus* are in red.
Genes from *Mnemiopsis leidyi* (Pang et al. 2010) are in blue.

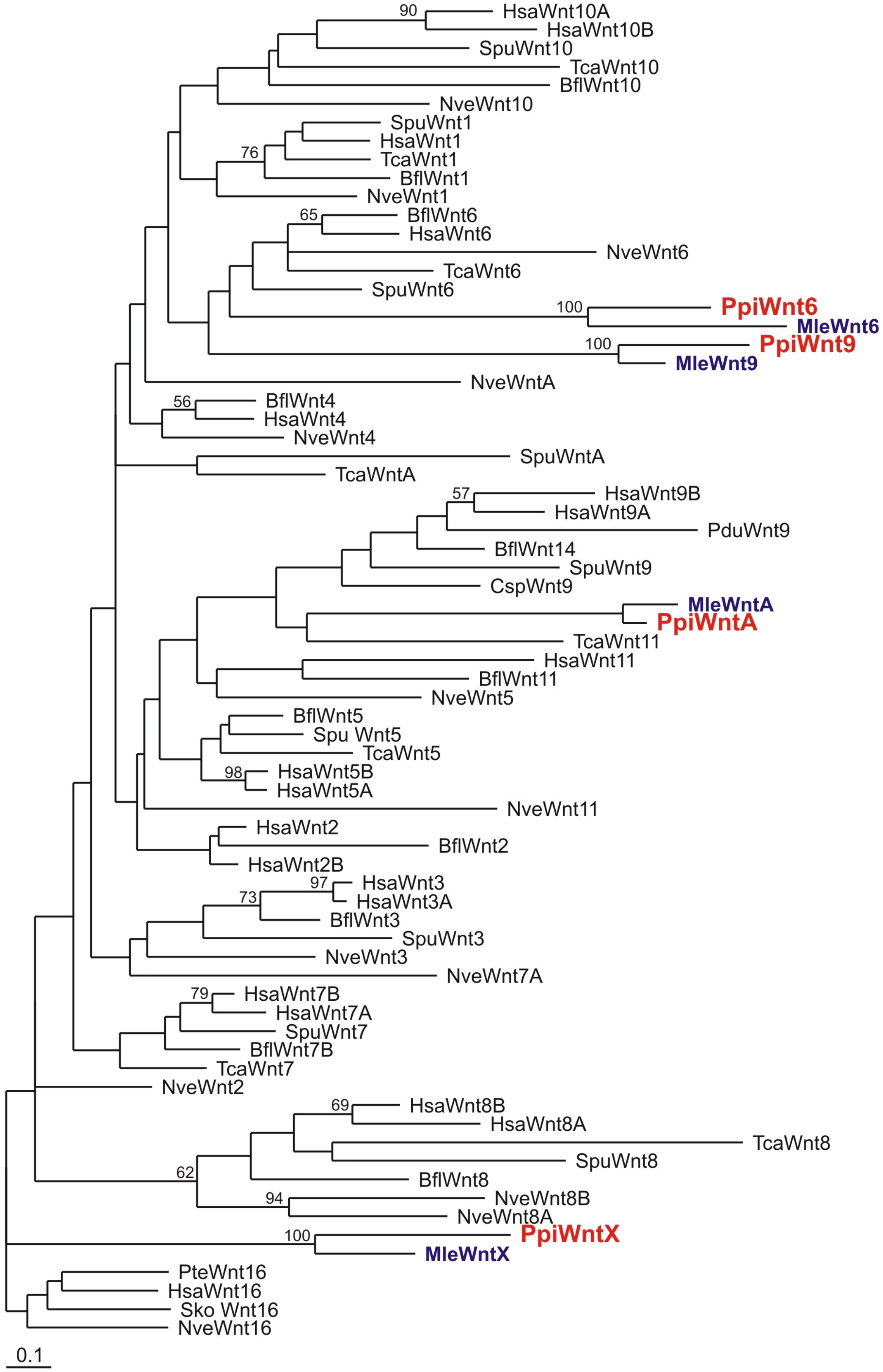
90
HsaWnt10A
HsaWnt10B
SpuWnt10
TcaWnt10
BflWnt10
NveWnt10
SpuWnt1
HsaWnt1
76
TcaWnt1
BflWnt1
NveWnt1
65
BflWnt6
HsaWnt6
NveWnt6
TcaWnt6
SpuWnt6
100
PpiWnt6
MleWnt6
100
PpiWnt9
MleWnt9
NveWntA
56
BflWnt4
HsaWnt4
NveWnt4
SpuWntA
TcaWntA
57
HsaWnt9B
HsaWnt9A
PduWnt9
BflWnt14
SpuWnt9
CspWnt9
MleWntA
PpiWntA
TcaWnt11
HsaWnt11
BflWnt11
NveWnt5
BflWnt5
Spu Wnt5
TcaWnt5
98
HsaWnt5B
HsaWnt5A
NveWnt11
HsaWnt2
BflWnt2
HsaWnt2B
97
HsaWnt3
73
HsaWnt3A
BflWnt3
SpuWnt3
NveWnt3
NveWnt7A
79
HsaWnt7B
HsaWnt7A
SpuWnt7
BflWnt7B
TcaWnt7
NveWnt2
69
HsaWnt8B
HsaWnt8A
TcaWnt8
SpuWnt8
62
BflWnt8
94
NveWnt8B
NveWnt8A
100
PpiWntX
MleWntX
PteWnt16
HsaWnt16
Sko Wnt16
NveWnt16
0.1

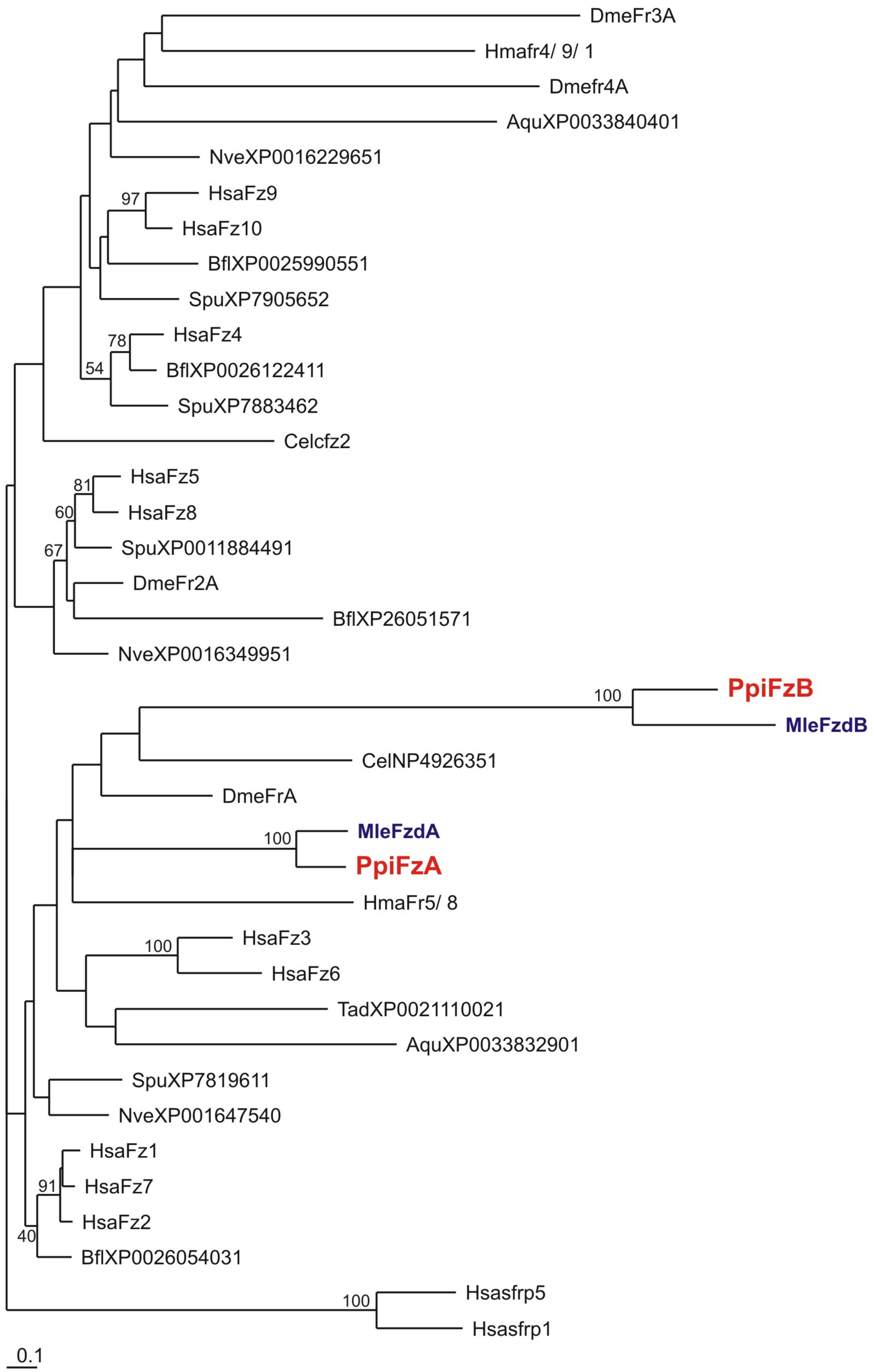
DmeFr3A
Hmafr4/ 9/ 1
Dmefr4A
AquXP0033840401
NveXP0016229651
97
HsaFz9
HsaFz10
BflXP0025990551
SpuXP7905652
78
HsaFz4
54
BflXP0026122411
SpuXP7883462
Celcfz2
81
HsaFz5
60
HsaFz8
67
SpuXP0011884491
DmeFr2A
BflXP26051571
NveXP0016349951
100
PpiFzB
MleFzdB
CelNP4926351
DmeFrA
100
MleFzdA
PpiFzA
HmaFr5/ 8
100
HsaFz3
HsaFz6
TadXP0021110021
AquXP0033832901
SpuXP7819611
NveXP001647540
HsaFz1
91
HsaFz7
HsaFz2
40
BflXP0026054031
100
Hsasfrp5
Hsasfrp1
0.1

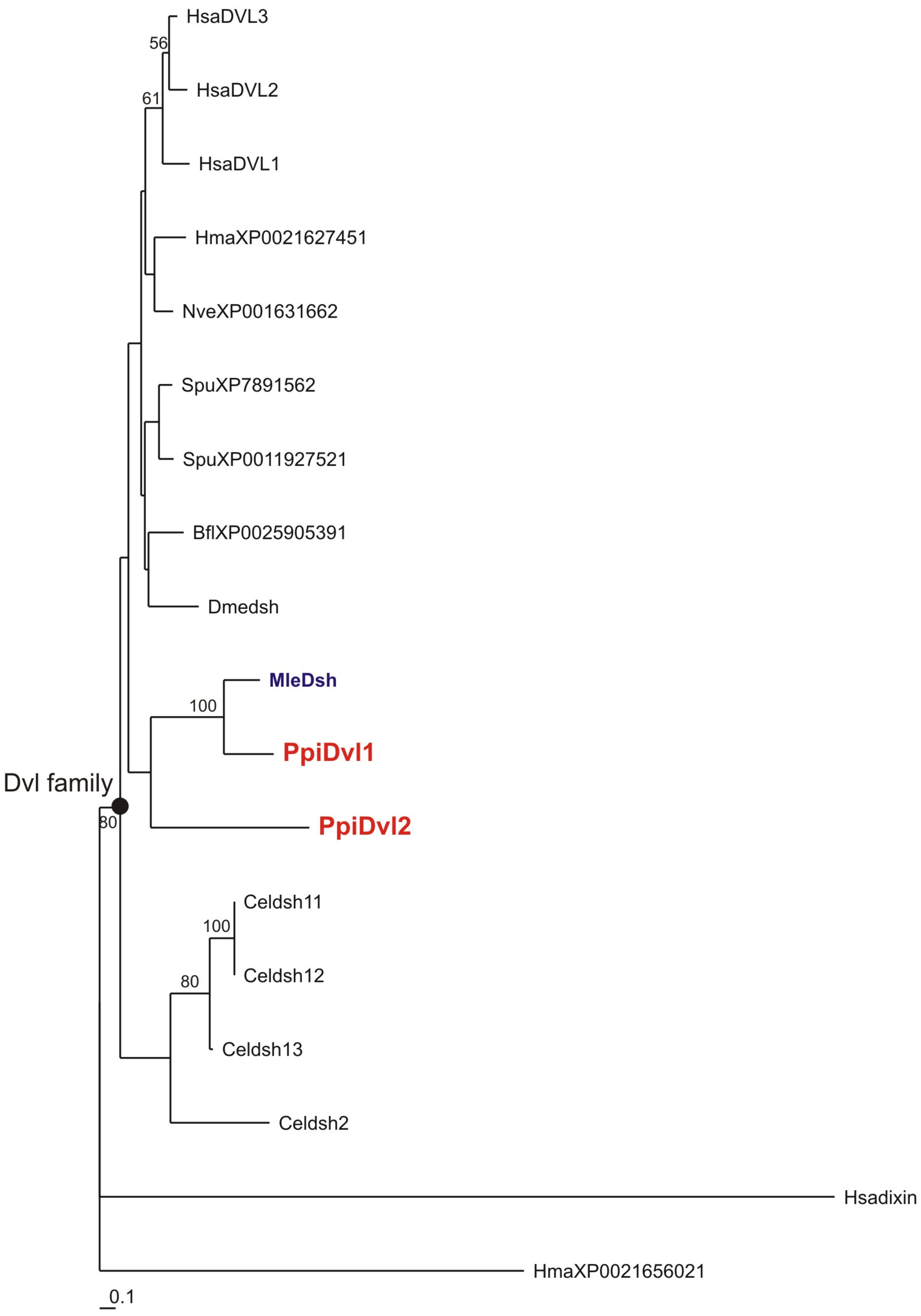
HsaDVL3
56
HsaDVL2
61
HsaDVL1
HmaXP0021627451
NveXP001631662
SpuXP7891562
SpuXP0011927521
BflXP0025905391
Dmedsh
MleDsh
100
PpiDvl1
Dvl family
PpiDvl2
80
Celdsh11
100
Celdsh12
80
Celdsh13
Celdsh2
Hsadixin
HmaXP0021656021
0.1

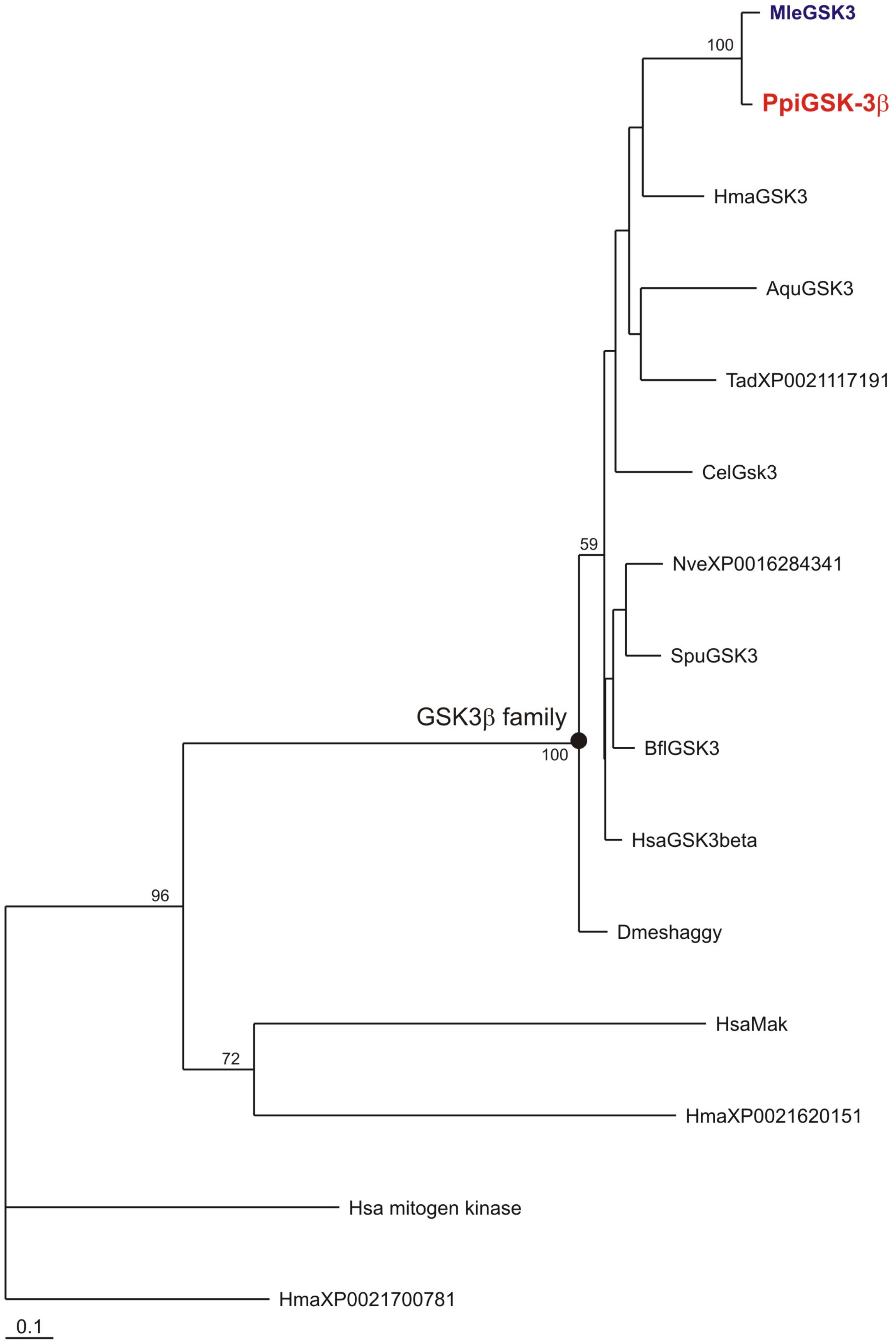
MleGSK3
100
PpiGSK-3β
HmaGSK3
AquGSK3
TadXP0021117191
CelGsk3
59
NveXP0016284341
SpuGSK3
GSK3β family
BflGSK3
100
HsaGSK3beta
96
Dmeshaggy
HsaMak
72
HmaXP0021620151
Hsa mitogen kinase
HmaXP0021700781
0.1

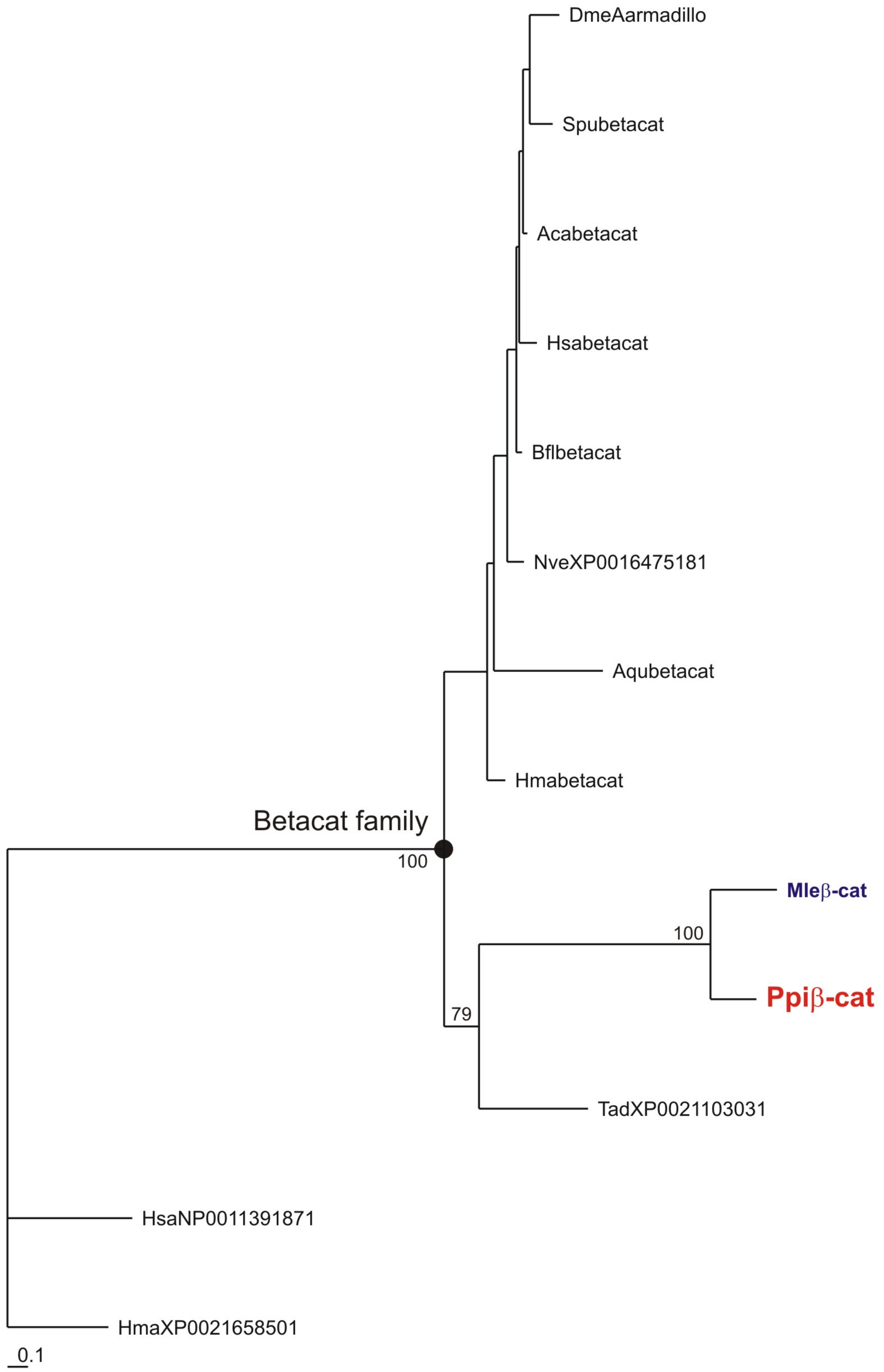
DmeAarmadillo
Spubetacat
Acabetacat
Hsabetacat
Bflbetacat
NveXP0016475181
Aqubetacat
Hmabetacat
Betacat family
100
Mleβ-cat
100
Ppiβ-cat
79
TadXP0021103031
HsaNP0011391871
HmaXP0021658501
0.1

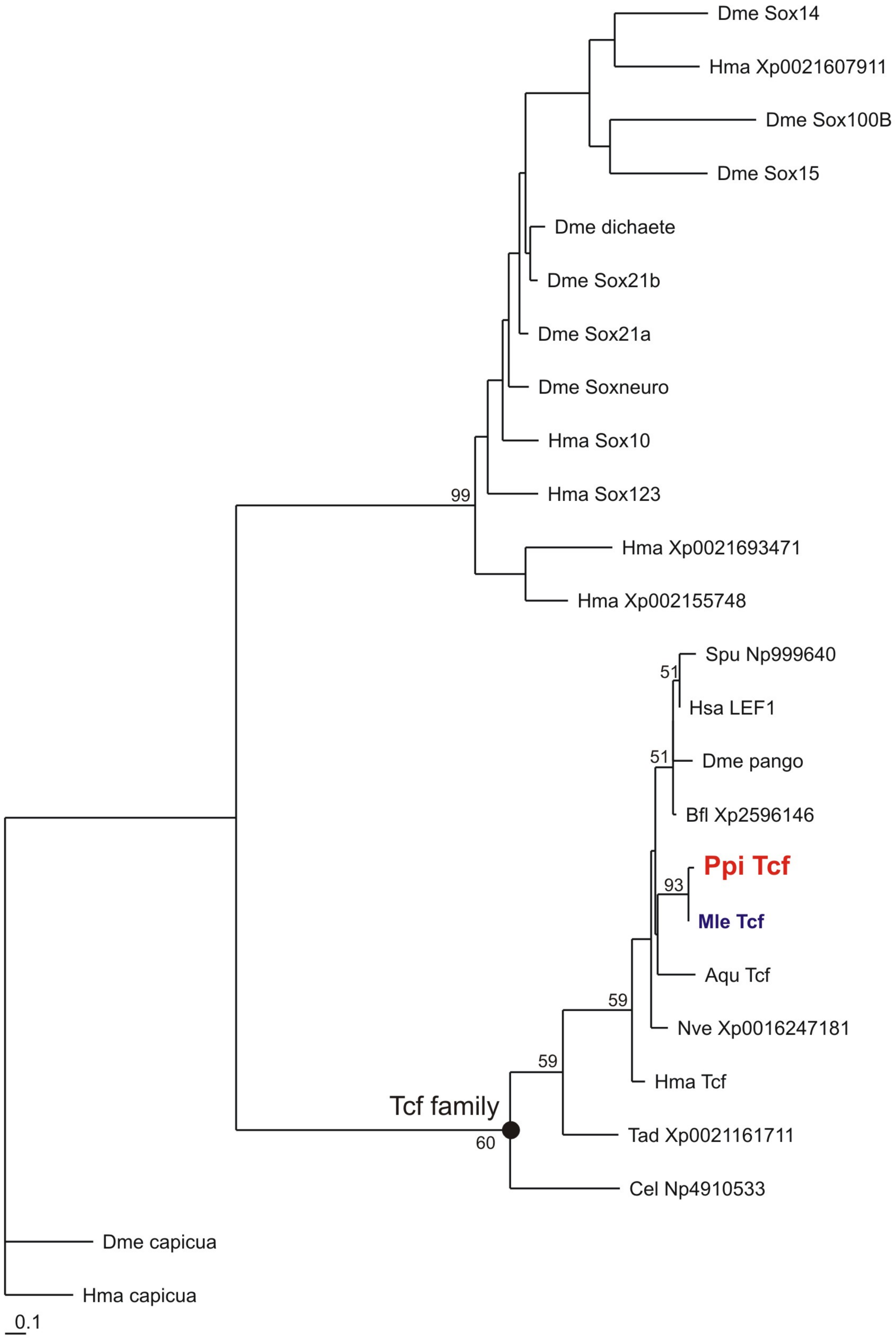
Dme Sox14
Hma Xp0021607911
Dme Sox100B
Dme Sox15
Dme dichaete
Dme Sox21b
Dme Sox21a
Dme Soxneuro
Hma Sox10
Hma Sox123
99
Hma Xp0021693471
Hma Xp002155748
Spu Np999640
51
Hsa LEF1
51
Dme pango
Bfl Xp2596146
Ppi Tcf
93
Mle Tcf
Aqu Tcf
59
Nve Xp0016247181
59
Hma Tcf
Tcf family
Tad Xp0021161711
60
Cel Np4910533
Dme capicua
Hma capicua
0.1
