# Supplementary material for: Evidence for Involvement of Wnt Signalling in Body Polarities, Cell Proliferation, and the Neuro-Sensory System in an Adult Ctenophore
Source: PLoS One. 2013 Dec 31;8(12):e84363. doi: 10.1371/journal.pone.0084363 (PMC3877318; doi:10.1371/journal.pone.0084363)

Evidence for involvement of Wnt signalling in body polarities, cell proliferation, and the neuro-sensory system in an adult ctenophore

Muriel Jager, Cyrielle Dayraud, Antoine Mialot, Eric Quéinnec, Hervé le Guyader and Michaël Manuel

Supporting Information File S2: Negative (sense probe) and positive (*PpiTbxE* and *PpiPax1*) controls for whole mount *in situ* hybridisation

The *PpiTbxE* gene is strongly expressed in the nurse cells of the female gonads. The *PpiPax1* gene is expressed throughout the meridional canals. None of them show any signal around the mouth. Scale bars: 1 mm.

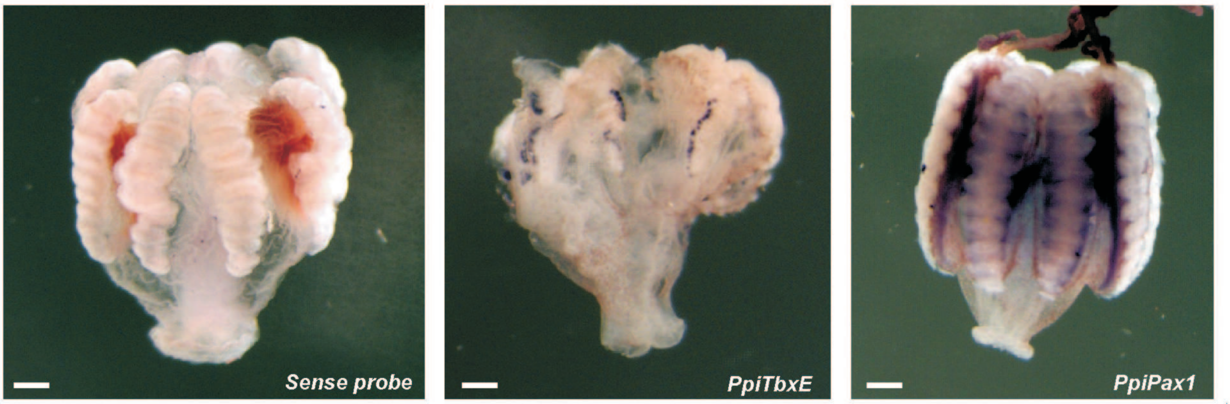

Supplement: File S2 — Negative (sense probe) and positive ( PpiTbxE and PpiPax1 ) controls for whole mount in situ hybridisation. (PDF) [file pone.0084363.s002.pdf]
