# Supplementary material for: Evidence for Involvement of Wnt Signalling in Body Polarities, Cell Proliferation, and the Neuro-Sensory System in an Adult Ctenophore
Source: PLoS One. 2013 Dec 31;8(12):e84363. doi: 10.1371/journal.pone.0084363 (PMC3877318; doi:10.1371/journal.pone.0084363)
Supplement: File S3 — DNA label incorporation experiments providing insights into cellular dynamics in the polar fields. (PDF) [file pone.0084363.s003.pdf]

Evidence for involvement of Wnt signalling in body polarities, cell proliferation, and the neuro-sensory system in an adult ctenophore

Muriel Jager, Cyrielle Dayraud, Antoine Mialot, Eric Quéinnec, Hervé le Guyader and Michaël Manuel

Supporting Information File S3: DNA label incorporation experiments providing insights into cellular dynamics in the polar fields

EdU 14h pulse, no chase

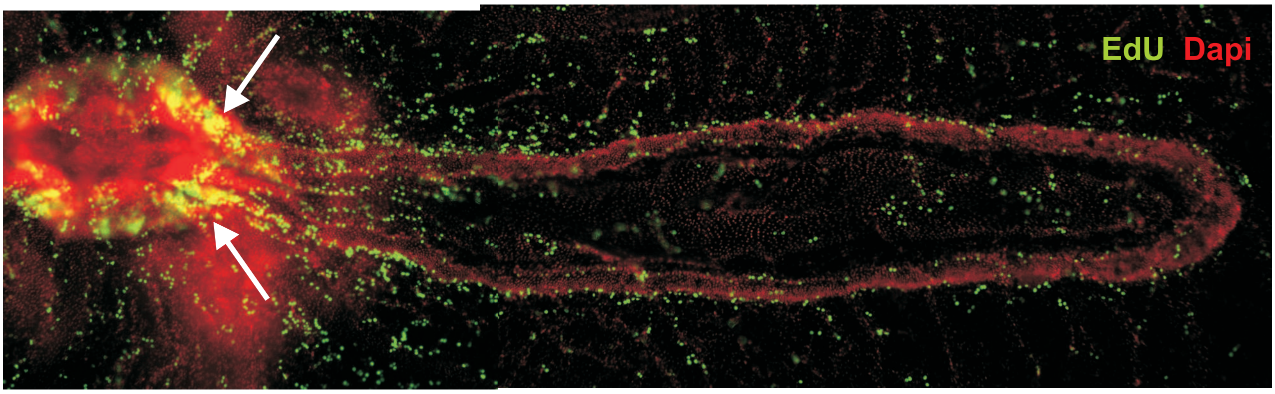

Nuclei having incorporated the DNA label are extremely numerous and concentrated at the proximal extremity of the polar field (arrows). Labelled nuclei are also present but much less concentrated in the proximal quarter of the polar field, and are even sparser in the rest of the polar field.

EdU 20h pulse, 5 days chase

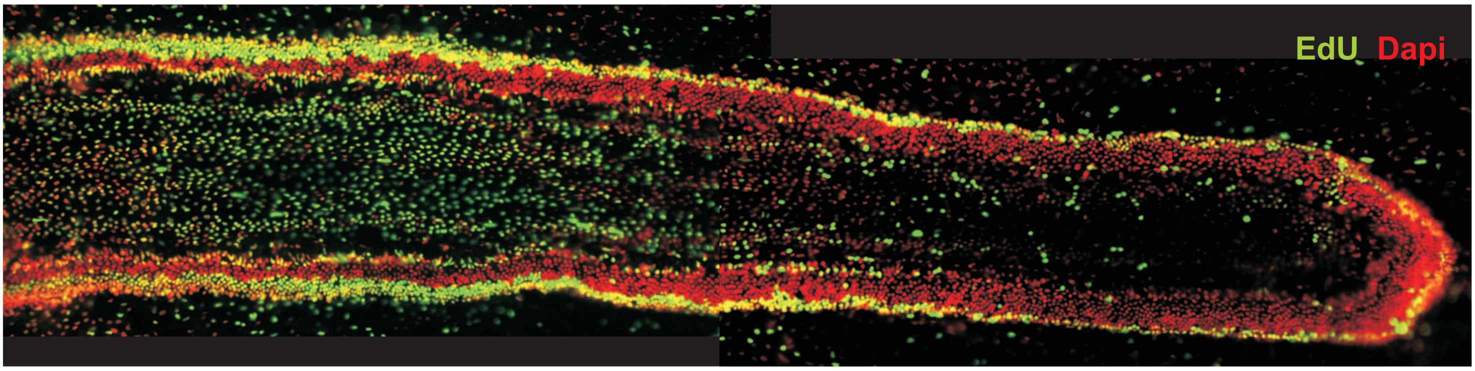

The apical organ (not shown) is located next to the left border of the picture. See Alié et al. (2011) for long-term retention of the DNA label in the four groups of putative stem cells at the polar field proximal extremities around the apical organ.
This picture shows the distribution at the scale of the whole polar field of labelled nuclei, five days after they have incorporated the label. During the chase, labelled cells (initially located at the proximal extremity, see above) have differentiated and meanwhile they have been displaced towards the distal pole. Cells of the left half (= proximal half) are clearly younger on average that cells of the right (distal) half.

Altogether these data demonstrate rapid and oriented cell renewal in the polar field, with new cells produced predominantly at the proximal extremity.
