# Supplementary material for: Evidence for Involvement of Wnt Signalling in Body Polarities, Cell Proliferation, and the Neuro-Sensory System in an Adult Ctenophore
Source: PLoS One. 2013 Dec 31;8(12):e84363. doi: 10.1371/journal.pone.0084363 (PMC3877318; doi:10.1371/journal.pone.0084363)

Evidence for involvement of Wnt signalling in body polarities, cell proliferation, and the neuro-sensory system in an adult ctenophore

Muriel Jager, Cyrielle Dayraud, Antoine Mialot, Eric Quéinnec, Hervé le Guyader and Michaël Manuel

Supporting Information File S4: Gene expression in the comb rows

This figure shows expression for each gene in about ten successive basal cushions along a given comb row. For *PpiFzA*, two different preparations are shown which differed by the duration of the revelation step. Scale bars: 100 μm.

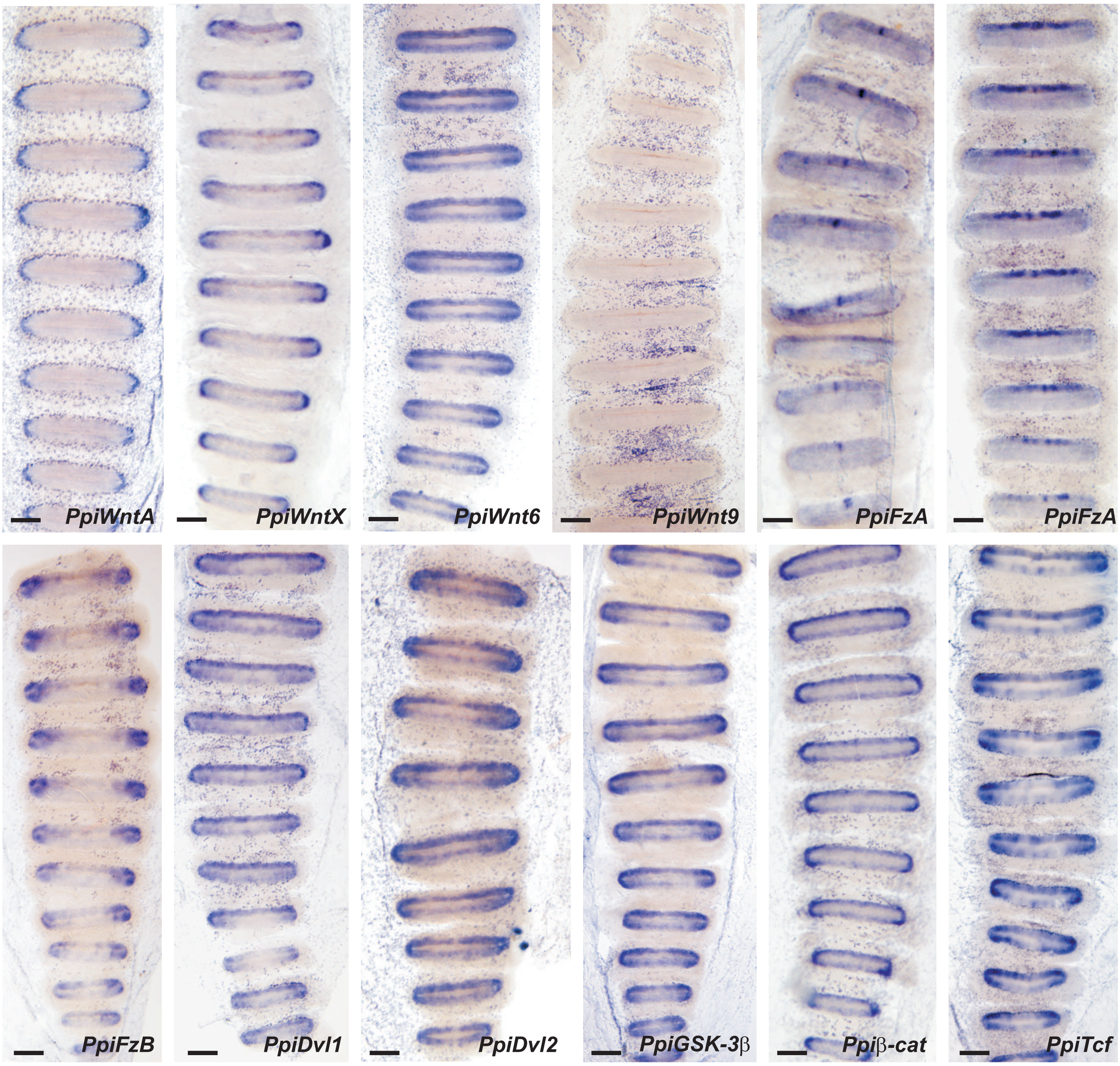

Supplement: File S4 — Gene expression in the comb rows. (PDF) [file pone.0084363.s004.pdf]
